# Secondary organic aerosol association with cardiorespiratory disease mortality in the United States

**DOI:** 10.1038/s41467-021-27484-1

**Published:** 2021-12-16

**Authors:** Havala O. T. Pye, Cavin K. Ward-Caviness, Ben N. Murphy, K. Wyat Appel, Karl M. Seltzer

**Affiliations:** 1 Office of Research and Development, U.S. Environmental Protection Agency, 109 TW Alexander Dr, Research Triangle Park, NC 27711 USA; 2 Office of Research and Development, U.S. Environmental Protection Agency, 104 Mason Farm Rd, Chapel Hill, NC 27514 USA; 3 Oak Ridge Institute for Science and Education Postdoctoral Fellow in the Office of Research and Development, U.S. Environmental Protection Agency, 109 TW Alexander Dr, Research Triangle Park, NC 27711 USA

**Keywords:** Environmental impact, Atmospheric chemistry, Atmospheric chemistry, Cardiovascular diseases

## Abstract

Fine particle pollution, PM_2.5_, is associated with increased risk of death from cardiorespiratory diseases. A multidecadal shift in the United States (U.S.) PM_2.5_ composition towards organic aerosol as well as advances in predictive algorithms for secondary organic aerosol (SOA) allows for novel examinations of the role of PM_2.5_ components on mortality. Here we show SOA is strongly associated with county-level cardiorespiratory death rates in the U.S. independent of the total PM_2.5_ mass association with the largest associations located in the southeastern U.S. Compared to PM_2.5_, county-level variability in SOA across the U.S. is associated with 3.5× greater per capita county-level cardiorespiratory mortality. On a per mass basis, SOA is associated with a 6.5× higher rate of mortality than PM_2.5_, and biogenic and anthropogenic carbon sources both play a role in the overall SOA association with mortality. Our results suggest reducing the health impacts of PM_2.5_ requires consideration of SOA.

## Abstract (web summary)

Fine particle air pollution causes premature death, but the role of different fine particle components in mortality is not well characterized. Here, the authors show the secondary organic aerosol component of fine particle mass is associated with significant cardiorespiratory mortality in the U.S.

## Introduction

Ambient fine particulate matter, PM_2.5_, is estimated to be responsible for up to 9 million deaths per year worldwide^1,2^, including 15 deaths per 100,000 people in the U.S^3^. Multiple studies show a link between exposure to PM_2.5_ and cardiovascular and respiratory (cardiorespiratory, CR) disease, hospital admissions, and mortality^4–8^. While less than ten components of PM_2.5_ contribute significantly to mass, PM_2.5_ includes about 50 easily identifiable inorganic species^9^ and potentially hundreds of thousands of individual organic compounds^10^. Total PM_2.5_ mass has been more consistently associated with adverse health outcomes than individual components^11^, potentially due to limited access to composition in historical epidemiological analyses.

Information on the role of specific sources of organic aerosol (OA) in adverse health outcomes is even more limited than for other components of PM_2.5_. Recent observations indicate that OA can exceed the mass of other common PM_2.5_ components such as sulfate^12^. Secondary organic aerosol (SOA) is produced from reactions of anthropogenic and natural emissions throughout the year^13,14^ and is a dominant component of total OA even in urban locations^15–17^. While ambient PM_2.5_ concentrations are expected to decline in the future as anthropogenic emissions are controlled, adverse health effects persist below current U.S. air quality standards^18^, and a relative increase in SOA could lead to increased health impacts per unit mass^19^.

In the southeastern U.S., emissions of monoterpenes and isoprene from vegetation mix with anthropogenic pollutants like nitrogen oxides (NO_x_) and other volatile organic compounds (VOCs) to form SOA in high concentration^13,20–22^. Underlying medical conditions like heart disease as well as CR disease mortality are also higher in the Southeast than the rest of the U.S. as a result of multiple socioeconomic and behavioral factors^23^. In addition, higher rates of stroke mortality in the stroke belt^24^ have persisted for decades^25^, and cardiovascular mortality rates have been slower to decline in the Southeast than in other regions of the U.S^23^. While previous air quality models struggled to reproduce measured organic carbon (OC), advances in algorithms for SOA formation pathways (oxidation of monoterpenes^13,22^, isoprene^20,26^, and anthropogenic volatile organic compounds^27^) have essentially eliminated OC underestimates in the current Community Multiscale Air Quality (CMAQ) model^28,29^. Improved SOA estimates combined with multi-decadal reductions of particulate sulfate concentrations allow for an examination of the role of SOA in CR mortality not previously possible. To the best of our knowledge, no epidemiological studies have considered the association of SOA, as estimated by any of the current generation of predictive algorithms, with health outcomes.

In this work, we use year 2016 age-adjusted CR disease mortality rate data from the Centers for Disease Control and Prevention (CDC) and predicted PM_2.5_ in a cross-sectional framework to associate county-level per capita mortality with ambient PM_2.5_ and its components while adjusting for a broad array of relevant confounders (see Methods section). We use multiple linear regression to examine both the contiguous United States as well as the southeastern U.S. for different granularity in OA composition information.

## Results

**Fig. 1:**
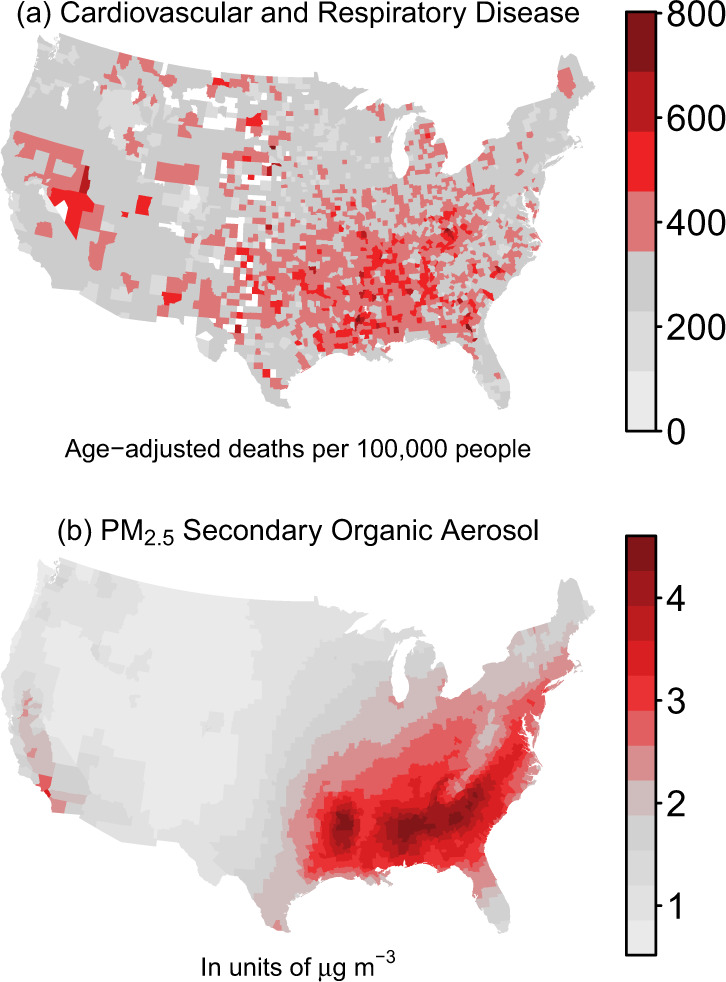
Cardiorespiratory disease mortality rates and secondary organic aerosol concentrations. County-level, year 2016 (**a**) cardiovascular and respiratory disease age-adjusted death rates (per 100,000 in population) are from CDC and (**b**) PM_2.5_ secondary organic aerosol concentrations are predicted by CMAQ. White in (**a**) indicates no death rate data while light gray indicates low reported rates.

**Fig. 2:**
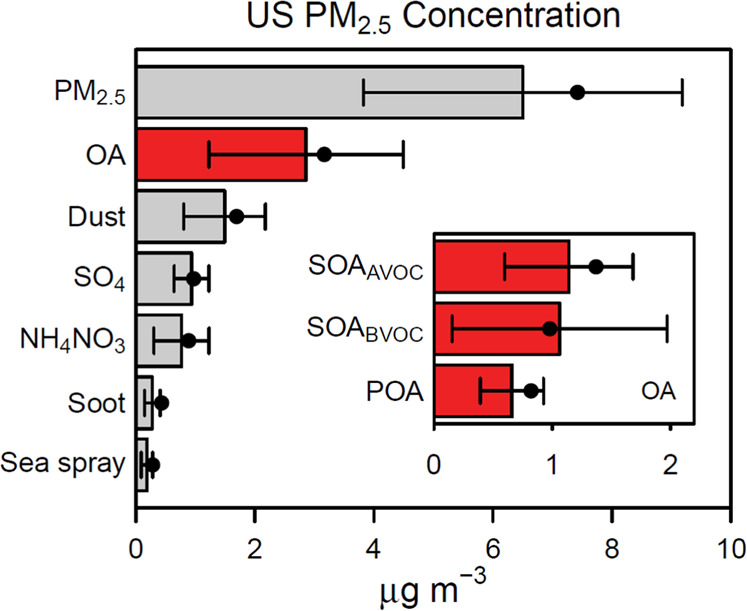
Contiguous U.S. county-level average predicted concentration of PM_2.5_ and its major components (bars) for 2016 (*n* = 2708 counties). Major components include organic aerosol (OA), a calcium-iron-silicon-aluminum rich aerosol (dust), sulfate (SO_4_), ammonium and nitrate (NH_4_NO_3_), an elemental carbon and potassium rich aerosol (soot), and a chloride-sodium-magnesium rich aerosol (sea spray). Inset in red are the subcomponents of OA: SOA from anthropogenic VOCs (SOA_AVOC_), SOA from biogenic VOCs (SOA_BVOC_), and primary organic aerosol (POA). Error bars represent ±1 IQR variation in the pollutant from the county-level average. Points represent population-weighted concentrations.

In 2016, CR disease in the U.S. was responsible for a county-level median of 320 age-adjusted deaths per 100,000 in population (2708 counties). The southeastern U.S. experienced a slightly higher rate of 360 age-adjusted deaths per 100,000 in population (646 counties) (Fig. 1a). The predicted county-wide, annual-average U.S. PM_2.5_ concentration was 6.5 µg m^−3^ with OA the most abundant major component at 2.9 µg m^−3^. Other major PM_2.5_ components included a calcium (Ca)-iron (Fe)-silicon (Si)-aluminum (Al)-rich dust, sulfate (SO_4_), ammonium and nitrate (NH_4_NO_3_), elemental carbon- and potassium (K)-rich soot, and chloride (Cl)-sodium (Na)-magnesium (Mg)-rich sea spray aerosol (Fig. 2, see also the Supplementary Information).

### PM_2.5_ SOA association with mortality

**Fig. 3:**
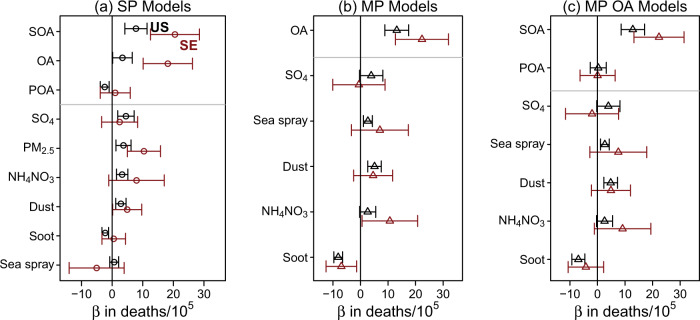
Association of PM_2.5_ and its components with death rates across the contiguous U.S. (*n* = 2708 counties) in black and southeastern (SE) U.S. (*n* = 646 counties) in red (open symbol) determined via regressed coefficients (β) from multiple linear regression and their 95% confidence intervals (whiskers). Models forms (see Methods, Table 2) are **a** single pollutant (SP, circles) or multipollutant (triangles) (**b**) for PM_2.5_ components (MP) and **c** with refinement of OA subcomponents (MP OA). Regressed coefficients correspond to IQR-normalized species concentrations (Supplementary Table 1) in units of deaths per 100,000 in population. Horizontal gray lines are used to visually separate results for OA and its subcomponents (primary organic aerosol, POA, and secondary organic aerosol, SOA) from the other components.

PM_2.5_, as well as multiple PM_2.5_ components, were strongly associated with CR mortality across the contiguous U.S. in 2016 (Fig. 3a). Variability in total PM_2.5_ (estimated by the inter-quartile range (IQR)) was associated with an increase of 3.7 (95% CI: 1.2–6.2) CR deaths per 100,000 people (Table 1), a 1.2% increase over the median CR death rate observed. For the southeastern U.S., an IQR increase in PM_2.5_ was associated with 9.1 (95% CI: 4.3–14) additional deaths per 100,000 people or a 2.8% increase over the median observed CR mortality. Associations of the CR death rate with IQR increases in the six primary PM_2.5_ components ranged from −2.3 to 4.5 across the contiguous U.S. and from −5.1 to 18 per 100,000 people across the southeastern U.S. in single pollutant models examining each component in a separate regression model with identical confounder adjustment (see Methods section and Supplementary Table 6).

**Table 1:** Regression model coefficients (β) and their 95% confidence intervals on a 1 µg m^−3^ and IQR-normalized basis for total PM_2.5_ (SP model), OA (MP model), and SOA (MP OA model) for the contiguous U.S. and southeastern U.S. Concentration is population weighted at the county level.

	*β* [95% CI] for 1 µg m^−3^ change in pollutant	*β* [95% CI] for IQR change in pollutant	Average concentration	IQR
	(deaths 10^−5^ µg^−1^ m^3^)	(deaths 10^−5^)	(µg m^−3^)	(µg m^−3^)
*Contiguous U.S.*				
PM_2.5_	1.4 [0.5, 2.3]	3.7 [1.2, 6.2]	7.4	2.7
OA	8.1 [5.4, 11]	13 [8.8, 18]	3.2	1.6
SOA	8.9 [6.0, 12]	13 [8.6, 17]	2.3	1.4
*Southeastern U.S.*				
PM_2.5_	9.1 [4.3, 14]	10 [5.0, 16]	7.9	1.1
OA	23 [13, 33]	22 [13, 32]	3.8	1.0
SOA	27 [16, 38]	22 [13, 31]	3.0	0.8

An IQR increase in the OA component of PM_2.5_ in μg m^−3^ (see Supplementary Table 1 for IQR values) was more strongly associated with CR mortality than was total PM_2.5_. The initial multipollutant (MP) model with six primary PM_2.5_ components indicated OA had the largest association by magnitude (*β* = 13, 95% CI: 8.8–18, deaths per 100,000) for an IQR change in concentration (Fig. 3b). Thus, an IQR increase in OA was associated with a greater elevation in county-level CR death rates than an IQR change in sulfate, ammonium and nitrate, sea spray, dust, or soot. When OA was further divided into primary organic aerosol (POA) and SOA (Fig. 3c), SOA (*β* = 13, 95% CI: 8.6–17, deaths per 100,000) but not POA (*β* = 0.29, 95% CI: −2.6–3.2, deaths per 100,000) was strongly associated with CR mortality. Similar results were obtained for the southeastern U.S. with SOA (*β* = 22, 95% CI: 13–31, deaths per 100,000 people) being more strongly associated with CR mortality than any other component and having a stronger association than observed in the entire U.S. The number of per capita deaths associated with IQR variability in SOA was 2.1 and 3.5 times larger than the number associated with total PM_2.5_ in the Southeast and contiguous U.S., respectively.

### Role of SOA beyond PM_2.5_ mass

To determine whether the association between SOA and CR mortality was independent of PM_2.5_ mass, additional models were adjusted for PM_2.5_ mass (SP-adj) or used residuals (R) resulting from the regression of SOA on PM_2.5_ mass as an exposure^30^ (see Methods section and Table 2). In the SP-adj framework, IQR variability in SOA was positively associated with mortality and had a larger regression coefficient than sulfate, ammonium and nitrate, sea spray, dust, soot, and POA as well as OA over both the contiguous and southeastern U.S. This indicates the association between SOA and CR mortality was not primarily driven by changes in total PM_2.5_. The SOA component residual was also associated with increased CR mortality rates across the U.S. and Southeast (Supplementary Fig. 4).

**Table 2:** Regression model forms.

Model	Form	Description
Single Pollutant (SP)	${{{\boldsymbol{D}}}}={\beta }_{0}+{\beta }_{1}{{{\boldsymbol{P}}}}{{{{\boldsymbol{M}}}}}_{{{{\boldsymbol{i}}}}}+\mathop{\sum }\limits_{j=1}^{N}{\beta }_{j}^{{{\hbox{'}}}}{{{{\boldsymbol{C}}}}}_{{{{\boldsymbol{j}}}}}$	Primary models evaluating association of PM_2.5_ or a component with mortality
Single Pollutant-Adjusted for total PM_2.5_ (SP-adj)	${{{\boldsymbol{D}}}}={\beta }_{0}+{\beta }_{1}{{{\boldsymbol{P}}}}{{{{\boldsymbol{M}}}}}_{{{{\boldsymbol{i}}}}}+{\beta }_{2}{{{\boldsymbol{P}}}}{{{{\boldsymbol{M}}}}}_{2.5}+\mathop{\sum }\limits_{j=1}^{N}{\beta }_{j}^{{{\hbox{'}}}}{{{{\boldsymbol{C}}}}}_{{{{\boldsymbol{j}}}}}$	SP model with adjustment of components for total PM_2.5_
Residual (R)	${{{\boldsymbol{D}}}}={\beta }_{0}+{\beta }_{1}{{{\boldsymbol{residua}}}}{{{{\boldsymbol{l}}}}}_{{{{\boldsymbol{i}}}}}+\mathop{\sum }\limits_{j=1}^{N}{\beta }_{j}^{{{\hbox{'}}}}{{{{\boldsymbol{C}}}}}_{{{{\boldsymbol{j}}}}}$ where ***residual***_***i***_ = ***PM***_***i***_ – *m*_*i*_ × ***PM***_***2.5***_ – *b*_*i*_ and *m*_*i*_ and *b*_*i*_ are component-specific coefficients obtained from the regression: ***PM***_***i***_ = *m*_*i*_ × ***PM***_***2.5***_ + *b*_*i*_	Second adjustment model of PM_2.5_ components for total PM_2.5_ based on residuals from regressing total PM_2.5_ components on PM_2.5_
Multipollutant (MP)	$$\begin{array}{c}{{{\boldsymbol{D}}}}={\beta }_{0}+\{{\beta }_{1}{{{\boldsymbol{P}}}}{{{{\boldsymbol{M}}}}}_{{{{\boldsymbol{OA}}}}}\}+{\beta }_{2}{{{\boldsymbol{P}}}}{{{{\boldsymbol{M}}}}}_{{{{\boldsymbol{Sea}}}}{{{\boldsymbol{spray}}}}}+{\beta }_{3}{{{\boldsymbol{P}}}}{{{{\boldsymbol{M}}}}}_{{{{\boldsymbol{Soot}}}}}\\ \qquad\quad\,+{\beta }_{4}{{{\boldsymbol{P}}}}{{{{\boldsymbol{M}}}}}_{{{{\boldsymbol{N}}}}{{{{\boldsymbol{H}}}}}_{4}{{{\boldsymbol{N}}}}{{{{\boldsymbol{O}}}}}_{3}}+{\beta }_{5}{{{\boldsymbol{P}}}}{{{{\boldsymbol{M}}}}}_{{{{\boldsymbol{S}}}}{{{{\boldsymbol{O}}}}}_{4}}+{\beta }_{6}{{{\boldsymbol{P}}}}{{{{\boldsymbol{M}}}}}_{{{{\boldsymbol{Dust}}}}}+\mathop{\sum }\limits_{j=1}^{N}{\beta }_{j}^{{\prime} }{{{{\boldsymbol{C}}}}}_{{{{\boldsymbol{j}}}}}\end{array}$$	Model with 6 primary PM_2.5_ components including OA to evaluate their independent associations
Multipollutant OA (MP OA)	$$\begin{array}{c}{{{\boldsymbol{D}}}}={\beta }_{0}+\{{\beta }_{1}{{{\boldsymbol{P}}}}{{{{\boldsymbol{M}}}}}_{{{{\boldsymbol{SOA}}}}}+{\beta }_{2}{{{\boldsymbol{P}}}}{{{{\boldsymbol{M}}}}}_{{{{\boldsymbol{POA}}}}}\}+{\beta }_{3}{{{\boldsymbol{P}}}}{{{{\boldsymbol{M}}}}}_{{{{\boldsymbol{Sea}}}}{{{\boldsymbol{spray}}}}}\\ \,+{\beta }_{4}{{{\boldsymbol{P}}}}{{{{\boldsymbol{M}}}}}_{{{{\boldsymbol{Soot}}}}}+{\beta }_{5}{{{\boldsymbol{P}}}}{{{{\boldsymbol{M}}}}}_{{{{\boldsymbol{N}}}}{{{{\boldsymbol{H}}}}}_{4}{{{\boldsymbol{N}}}}{{{{\boldsymbol{O}}}}}_{3}}+{\beta }_{6}{{{\boldsymbol{P}}}}{{{{\boldsymbol{M}}}}}_{{{{\boldsymbol{S}}}}{{{{\boldsymbol{O}}}}}_{4}}\\ \!\!\!\!\!\!\!\!\!\!\!\!\!\!\!\!\!\!\!\!\!\!\!\!\!\!\!\!\!\!\!\!\!\!\!\!\!\!\!\!\!\!+{\beta }_{7}{{{\boldsymbol{P}}}}{{{{\boldsymbol{M}}}}}_{{{{\boldsymbol{Dust}}}}}+\mathop{\sum }\limits_{j=1}^{N}{\beta }_{j}^{{\prime} }{{{{\boldsymbol{C}}}}}_{{{{\boldsymbol{j}}}}}\end{array}$$	Multipollutant composition model as in MP but with refinement of OA (OA subdivided into SOA and POA)
Multipollutant SOA (MP SOA)	$$\begin{array}{c}{{{\boldsymbol{D}}}}={\beta }_{0}+\big\{{\beta }_{1}{{{\boldsymbol{P}}}}{{{\boldsymbol{M}}}}_{{{{\boldsymbol{SO}}}}{{{\boldsymbol{A}}}}_{{{\boldsymbol{BVOC}}}}}+{\beta }_{2}{{{\boldsymbol{P}}}}{{{\boldsymbol{M}}}}_{{{{\boldsymbol{SO}}}}{{{\boldsymbol{A}}}}_{{{\boldsymbol{AVOC}}}}}\\ \qquad\qquad+{\beta }_{3}{{{\boldsymbol{P}}}}{{{\boldsymbol{M}}}}_{{{{\boldsymbol{POA}}}}}\big\}+{\beta }_{4}{{{\boldsymbol{P}}}}{{{\boldsymbol{M}}}}_{{{{\boldsymbol{Sea}}}}{{{\boldsymbol{spray}}}}}+{\beta }_{5}{{{\boldsymbol{P}}}}{{{\boldsymbol{M}}}}_{{{{\boldsymbol{Soot}}}}}\\ \qquad\qquad\qquad\;\;+{\beta }_{6}{{{\boldsymbol{P}}}}{{{\boldsymbol{M}}}}_{{{{\boldsymbol{N}}}}{{{\boldsymbol{H}}}}_{4}{{{\boldsymbol{N}}}}{{{\boldsymbol{O}}}}_{3}}+{\beta }_{7}{{{\boldsymbol{P}}}}{{{\boldsymbol{M}}}}_{{{{\boldsymbol{S}}}}{{{\boldsymbol{O}}}}_{4}}+{\beta }_{8}{{{\boldsymbol{P}}}}{{{\boldsymbol{M}}}}_{{{{\boldsymbol{Dust}}}}}+\mathop{\sum }\limits_{j=1}^{N}{\beta }_{j}^{\prime} {{{\boldsymbol{C}}}}_{{{\boldsymbol{j}}}}\end{array}$$	Multipollutant composition model as in MP OA but with refinement of SOA into SOA from oxidation of VOCs that are predominantly biogenic (BVOC) and anthropogenic (AVOC) in origin.

D, the outcome, is the age-adjusted cardiovascular and respiratory disease death rate in number of deaths per 100,000 people. By default, PM_i_ are concentrations of component *i* in μg m^−3^ normalized to the IQR, also in μg m^−3^, of that pollutant. When coefficients are presented per unit mass, the IQR normalization does not occur. PM_2.5_ is total PM_2.5_ concentration. ${{{\rm{\beta }}}}$ values indicate regressed coefficients. Confounders (N = 28 by default) with values, C_j_, are included in each model. Bold indicates a vector of county-level values. In MP models, brackets are placed around OA components for emphasis.

SOA was also associated with higher CR mortality rates on a 1 µg m^−3^ mass basis. Across the contiguous U.S., SOA was associated with 8.9 (95% CI: 6.0–12) additional deaths per 100,000 in population for a 1 µg m^−3^ increase in concentration indicating about 6.5 × higher association than total PM_2.5_ (see Table 1). In the southeastern U.S., SOA was associated with 27 (95% CI: 16–38) additional deaths per 100,000 in population for a 1 µg m^−3^ increase in concentration or about a 3.0× higher association than PM_2.5_. A 1 µg m^−3^ change in SOA in the southeastern U.S. also showed a 3.0× higher association with per capita CR mortality than for nationwide conditions. For 1 µg m^−3^ of PM_2.5_, the association was 6.6× higher in the Southeast than across the U.S.

### Role of SOA subcomponents

**Fig. 4:**
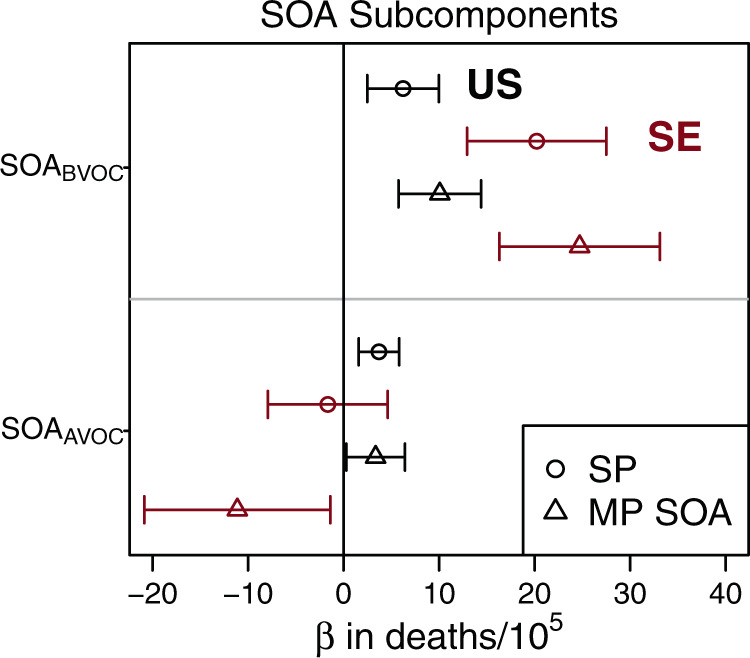
Association of PM_2.5_ SOA subcomponents with death rates across the contiguous U.S. (*n* = 2708 counties) in black and southeastern (SE) U.S. (*n* = 646 counties) in red (open symbol) determined via regressed coefficients (*β*) from multiple linear regression and their 95% confidence intervals (whiskers). Model forms are single pollutant (SP, circles) and multipollutant with refinement of SOA subcomponents (MP SOA, triangles). SOA_BVOC_ and SOA_AVOC_ are from the oxidation of VOCs that are predominantly biogenic and anthropogenic in origin, respectively. Regressed coefficients correspond to IQR-normalized species concentrations in units of deaths per 100,000 in population.

A multipollutant model (MP SOA) was used to estimate the health effects of SOA subcomponents including SOA from VOCs that were predominantly biogenic in origin (SOA_BVOC_) versus those that were anthropogenic in origin (SOA_AVOC_). SOA_BVOC_ showed a positive association with death rates across the U.S. (*β* = 10, 95% CI: 5.8–14, deaths per 100,000 people for the contiguous U.S. per IQR change, IQR = 0.90 µg m^−3^) as well as in the southeastern U.S. (*β* = 25, 95% CI: 16–33, deaths per 100,000 people per IQR change; IQR = 0.72 µg m^−3^) (Fig. 4, MP SOA). Sensitivity models on both the contiguous and southeastern U.S. domains showed SOA from biogenic VOCs was associated with CR mortality even after adjustment for total PM_2.5_, although the SOA_BVOC_-CR mortality association depended on the PM_2.5_ adjustment method (Supplementary Fig. 4). Further subdivision of SOA_BVOC_ into subcomponents representing the SOA from oxidation of terpenes and isoprene also showed positive associations of each subcomponent with CR mortality; however, relationships varied among model frameworks (Supplementary Figs. 4 and 5), and terpene- versus isoprene-derived SOA may be difficult to robustly separate due to correlation between them on a nationwide basis (*r* = 0.79).

Anthropogenic-VOC derived SOA was slightly more abundant than SOA_BVOC_ both nationwide (Fig. 2, inset) and in the southeastern U.S. with locations near Los Angeles, Atlanta, New York, and other cities showing local maximums in concentration. Multipollutant model results indicated higher levels of anthropogenic SOA were associated with higher county-level CR mortality rates across the contiguous U.S. (*β* = 3.4, 95% CI: 0.29–6.4, deaths per 100,000 people per IQR change) but indicated an inverse (*β* = −11, 95% CI: −21 to −1.4, deaths per 100,000 people per IQR change) association with death rates for the southeastern U.S. (Fig. 4, MP SOA). Sensitivity modeling showed the region of interest (contiguous vs southeastern U.S.) affected the direction of association for SOA_AVOC_.

### Utility of observed OC

OC is the only routinely measured indicator of organic PM_2.5_ at EPA Air Quality System (AQS) sites. Predicted OC was highly correlated with OA and SOA from the model (Pearson *r* = 0.97 or higher). Like OA, increases in model-predicted OC for the contiguous U.S. (2708 counties), showed a positive association with CR mortality (*β* = 2.3, 95% CI: −1.1–5.6, deaths per 100,000 people per IQR change, IQR = 0.92 µg m^−3^). After considering the role of PM_2.5_ mass (SP-adj or R model framework), the association of model-predicted OC with CR mortality remained positive for the southeastern U.S. only. Sub-setting the complete contiguous U.S. model-predicted OC (*n* = 2708) to the AQS sites with data for 2016 (n = 232), resulted in no association between predicted OC and increased CR mortality rates (*β* = −1.3, 95% CI: −12–9.3, deaths per 100,000 people per IQR change, IQR = 1.2 µg m^−3^). Measured OC at AQS sites indicated an association between observed OC and county-level CR mortality rates across the U.S. (*β* = 4.1, 95% CI: 1.1–7.0, deaths per 100,000 people per IQR change, IQR = 1.2 µg m^−3^), suggesting, measured OC has greater power (smaller confidence interval with larger magnitude association) than modeled OC in a direct comparison.

## Discussion

This work demonstrates a robust association between annual-average SOA concentrations and county-level CR death rates in a cross-sectional study. Associations were elevated in the southeastern U.S. as compared to the contiguous U.S. Not only was SOA associated with higher CR death rates than other components considered, it showed a larger association per unit mass than total PM_2.5_. The role of SOA was separately distinguished from that of total PM_2.5_ mass as indicated by single pollutant adjusted for PM_2.5_ and component residual model results.

Laboratory experiments indicate a role for SOA in adverse health outcomes. SOA exposure has been shown to result in lung cell death^31^ and formation of reactive oxygen species that can lead to adverse health effects^32–35^. Acellular assays of laboratory-generated SOA from terpenes, isoprene, and aromatics show oxidative potential that can approach that of ambient PM_2.5_ and known hazardous air pollutants such as diesel exhaust particles^35–38^. Continued atmospheric processing, including oxidation of SOA or POA also leads to increased oxidative potential^35,39^. In addition, exposure to isoprene-derived SOA has been shown to modify gene expression in human airway epithelial cells^40^. CR mortality associations with SOA in this work were not driven by a single SOA subcomponent and a consensus on which SOA systems drive redox reactivity as determined by dithiothreitol acellular assays is also lacking^34^. However, Bates et al.^35^ indicate the highest oxidative potential for aromatic (predominantly anthropogenic) SOA followed by terpene then isoprene SOA. Combustion sources, like biomass burning and vehicles which lead to both primary and secondary OA, are likely a significant source of oxidative potential in urban areas^41^ and have been linked with pediatric respiratory disease emergency visits in the eastern U.S.^42^.

Anthropogenic SOA is more abundant than biogenic SOA and POA in locations like the Northeast and California (Fig. 2 and Supplementary Fig. 1j–l), but it did not have a higher association with CR mortality when compared to other SOA subcomponents and showed no association when analysis was restricted to the southeastern U.S. Anthropogenic SOA model algorithms are generally more empirical than for terpene and isoprene SOA pathways. In addition, significantly less data is available from urban atmospheres in the U.S. that could be used to better elucidate chemical pathways of anthropogenic SOA. Therefore, the mixed associations for anthropogenic SOA on U.S. vs southeastern U.S. domains should be revisited as model representations continue to improve or as observational data sets for similar analysis become available. Biogenic SOA was robustly associated with higher CR mortality. While the ability to separate biogenic SOA into further subcomponents may be limited due to correlation, where correlation between the isoprene and monoterpene SOA was lowest (in the Southeast, *r* = 0.54) both pollutants showed positive associations with CR mortality.

Future work should continue to quantify the effects of different PM components on health, with consideration of SOA and its subcomponents. Historically, the association of individual PM_2.5_ components and health effects have lacked consistency, and as a result, regulation of PM_2.5_ in the U.S. has focused on total mass^11^. SOA concentration measurements are not routinely available, so the analysis shown here relied upon the ability of a model to robustly predict SOA. New data sets of measured SOA concentrations could be directly used in health impact assessment and lead to improved air quality models which will also ultimately lead to refinement of health effect estimates. An improved understanding of SOA health implications could influence control strategies to effectively reduce mortality. For example, isoprene SOA is likely best reduced by targeting sulfur oxide emissions^20^ while monoterpene SOA can be reduced via controls on NO_x_^21,22^. In addition, an improved representation of SOA from anthropogenic sources could improve health impact estimates and mitigation by providing insight into which sources (vehicle, evaporative chemical products, commercial and residential cooking, solid fuel burning, etc.) most affect public health.

This study benefited from having a large dataset spanning 2708 counties across the U.S. and high-quality air pollutant concentration data. The model algorithms and processes responsible for SOA prediction were previously evaluated against data from field campaigns^13,26,27,43^ as well as nationwide OC observations^27,29^. This allowed for a systematic examination of PM_2.5_ components, specifically SOA, that is not standardly available. In addition, results from initial statistical models were examined through sensitivity simulations confirming the association between SOA and CR mortality. However, the analysis performed here does not exclude a role for components other than SOA from being important for health. Primary particle effects may be acute and on smaller spatio-temporal scales than examined here. Future work focused on individual health outcomes and exposures, rather than county-level information, could better characterize near-source exposure and consequent health implications, which may be more relevant for some components and communities^44^.

The analysis here using measured OC is consistent with SOA showing strong association with CR mortality. The ability of measured OC to show a more precise relationship with cardiorespiratory mortality than modeled OC suggests that increasing the spatial coverage of OC measurements could be beneficial for understanding the health impacts of PM_2.5_ components, particularly in the southeastern U.S. where the limited availability of AQS sites prohibited an observation-based analysis. Total OC lacks the distinction between POA and SOA which could explain why both positive and negative associations of OC with adverse health endpoints have been found in previous work^11,45,46^. However, new observations could employ online techniques^47^ to mitigate filter artifacts^48^, and measurement of specific chemical fragments^47^ or functional groups^49^ could lead to a better understanding of the role of OA subcomponents. Multiple studies have shown that combining air quality models and measurements together will likely provide the most robust information going forward^18,50^. As new SOA exposure estimates are created for additional years and through other techniques, similar analysis should be performed to further examine the role of SOA.

Health benefits due to changes in fine particle concentrations are quantified for multiple purposes including evaluation of the public health benefits of pollution reduction efforts^51^, co-benefits of carbon pricing^52^, effectiveness of emission controls^53^, and forecasting air quality impacts under future scenarios^54^. In each case, the mortality-related impacts of PM_2.5_ are influenced by changes in the total as dictated by the changes in individual components^55^. SOA is often explicitly excluded in these analyses^52,56,57^. The work here suggests a focus on primary emissions or inorganic components alone is insufficient to properly estimate health impacts of changes in PM_2.5_. If recent trends from the past decade continue^58,59^, sulfate will continue to decrease as a fraction of PM_2.5_ in favor of OA. Since SOA is an important driver of the health impacts of PM_2.5_ examined here, strategies to further reduce health impacts of PM_2.5_ via emission controls, as well as understanding how death rates have changed in the recent past, requires consideration of SOA.

## Methods

This cross-sectional study used multiple linear regression to estimate how spatial variability in pollutant concentrations is associated with CR death rates while adjusting for a wide array of confounders. Multiple sensitivity analyses were undertaken to understand the robustness of the associations and independence of estimated health effects.

### Pollutant concentrations

County-level pollutant concentrations (full description in Supplementary Table 2) were created by mapping 12 km by 12 km horizontal resolution CMAQ v5.3.1^28,29^ (Supplementary Table 8) predictions for 2016 by grid cell center latitude and longitude to county locations using the R (v3.6.2) libraries sp^60^, maps^61^, and maptools^62^. When multiple CMAQ cell centers fell within one county, all respective CMAQ cells were averaged to create the county-level prediction. Four small counties did not contain any CMAQ grid cell center. These predictions were created by identifying the most populous city in each of the four counties^63^ and the CMAQ grid cell containing that city. CMAQv5.3.1 tends to be relatively unbiased in its prediction of annual-average PM_2.5_ and OC on a nationwide basis (<10% absolute normalized mean bias compared to AQS observations; Supplementary Table 9 and Supplementary Fig. 6). Predictions for specific OA systems have been evaluated in previous work (summarized in Supplementary Table 10)^13,26,27^, and predictions from v5.3.1 for the summer in the Southeast are greatly improved over previous model versions.

Total OC in PM_2.5_ measured at Chemical Speciation Network (CSN) and the Interagency Monitoring of Protected Visual Environments (IMPROVE) network sites during 2016 was obtained from the EPA AQS. Both CSN and IMPROVE use a thermal optical reflectance method to measure OC collected on filters and report similar results for collocated samplers (12% bias)^64^. Observed OC data were aggregated to an annual average and multiple sites within a county were averaged into one county-level value for pairing with the health outcome. CMAQ OC predictions were determined by converting concentrations of OA by model species to OC using species-specific organic matter to OC ratios^13,26,27,43^.

### Cardiorespiratory deaths

The outcome of interest was year 2016 age-adjusted CR mortality rates as determined by the Centers for Disease Control and Prevention^65^. Age-adjusted rates are weighted by the population age relative to U.S. standard population and created by the CDC^66^. CR mortality was determined via ICD-10 codes I00-I99 (diseases of the circulatory system) and J00-J98 (diseases of the respiratory system). All I00-J98 deaths, rather than specific disease outcomes such as stroke, were used to increase sample size and power, particularly in low-population rural areas where deaths for specific causes can be very low and thus poorly estimated. Counties with missing death rates (CDC does not report rates when the count is below 9) were removed from the analysis. The CDC flagged counties with 20 or fewer CR deaths as having unreliable rates due to increased variance in the numerator. These counties remained in the analysis to increase the nationwide representativeness despite the potential for increased noise. County population was included as a weight in all models to better account for the precision in estimating rates (higher population counties naturally have more precision in estimating mortality rates) and account for the increased noise associated with low-population counties. In addition to the contiguous United States (48 states), subgroup analysis was performed on the southeastern U.S., defined as Kentucky, Tennessee, North Carolina, Mississippi, Alabama, Georgia, South Carolina, and Florida (EPA Region 4, https://www.epa.gov/aboutepa/visiting-regional-office).

### Confounders

County-level confounders including population demographics (e.g. race, sex, age), behaviors (e.g. smoking, obesity), social and economic factors (e.g. education, income, unemployment, insured adults), and environmental parameters (water quality, relative humidity, temperature) were included in all statistical models. Relative humidity and temperature were obtained from WRF v4.1.1 predictions and processed through the CMAQ modeling system including the Meteorology-Chemistry Interface Processor^67^ as in the work of Appel, et al.^29^. All other confounders were obtained from the University of Wisconsin Population Health Institute’s (UWPHI) County Health Rankings (CHR)^68^ 2018 dataset^69^. From the full set of CHR measures, metrics that were duplicative (e.g. demographic metrics that summed to one), a health outcome (e.g. poor health, life expectancy), or likely irrelevant to the outcome (e.g. driving alone, dentists, teen births) were removed from consideration. In addition, we only considered CHR factors with values for >95% of the contiguous U.S. counties. Combined with relative humidity and temperature, this resulted in 28 confounders. Underlying inputs to the UWPHI data come from the CDC, Bureau of Labor Statistics, American Community Survey, Census Bureau, Department of Agriculture, Centers for Medicare and Medicaid Services as well as other data sources and were generally representative of 2016 information. Supplementary Table 3 provides the full list of confounders along with their original data source and year represented. The statistical model input dataset, including predicted composition, outcome, and confounders, resulted in complete information for 2708 (out of 3075) counties for the contiguous U.S. and 646 (out of 736) counties for the southeastern U.S. The minimum, mean, maximum, and IQR for all confounders are summarized in Supplementary Table 1.

### Statistical models

Primary analyses use multiple linear regression to associate PM_2.5_ with CR mortality while adjusting for the confounders described above. County-level population (American Community Survey 5-year estimate) was included as a weight in each model as mentioned previously. In addition to total PM_2.5_, PM_2.5_ components were analyzed. Individual PM_2.5_ species estimated by CMAQ were grouped into six main components, mutually exclusive of each other and summing to total PM_2.5_: OA, sea spray, soot, NH_4_NO_3_, SO_4_, and dust. These components were created because many individual PM_2.5_ species are highly correlated (Supplementary Table 4) which limits the ability of statistical models to separate their individual health associations. The six main components had correlations with each other that did not exceed 0.67 (Pearson *r*, Supplementary Table 4). The conversion from raw CMAQ output to model components used in this work (Supplementary Table 2) along with the spatial distribution of concentrations (Supplementary Fig. 1) is available in the supplementary information.

In the second stage of models, the associations of PM_2.5_ components with mortality were adjusted for total PM_2.5_ both by including a term for PM_2.5_ within the models (SP-adj) and by regressing each component on total PM_2.5_ and taking the residual^30^ for the component variation that was not due to total PM_2.5_ variation (R) (Table 2). Since residual models remove the portion of the pollutant that is correlated with total PM_2.5_, any statistically significant positive association of the residual with the outcome indicates that variability in that component specifically contributes to the adverse health effect beyond its role in PM_2.5_ total mass.

To further explore the relationship between the components, a series of analyses included multiple components within a single model and primarily focused on whether associations with organic PM_2.5_, and further subdivisions of organic PM_2.5_, were independent of other PM_2.5_ components. These multipollutant models were constructed to provide increasing granularity of information on associations between SOA and SOA subcomponents. The first multipollutant composition model (MP) included OA as well as the five other major PM_2.5_ components in a single model. The second composition model (MP OA) further divided OA into POA and SOA. The correlation between PM_2.5_ components including POA and SOA did not exceed *r* = 0.79. The third, composition model (MP SOA) further divided SOA into two subcomponents based on the dominant origin (biogenic vs anthropogenic) of their precursor VOCs. SOA from C_10_–C_15_ terpenes as well as isoprene and related (e.g. glyoxal) species was labeled as SOA_BVOC_ while SOA from anthropogenic VOCs was labeled SOA_AVOC_ (Supplementary Table 2). The models are described in Table 2 and Supplementary Fig. 2.

Splines^70^ as implemented in generalized additive models (R package mgcv^71^) were used to construct concentration-response curves for the association between PM_2.5_ and county-level cardiorespiratory mortality rate (see Supplementary Fig. 3). All statistical models used R v4.0.0^72^. All regressed coefficients are presented per IQR increase in the exposure along with a 95% confidence interval unless otherwise indicated.

### Reporting summary

Further information on research design is available in the Nature Research Reporting Summary linked to this article.

## Supplementary information

Supplementary Information

Reporting Summary

## Data Availability

The complete set of county-level concentrations and multiple regression results generated in this study have been deposited at 10.5281/zenodo.5713903. Mortality rate data used in this work are available from the Centers for Disease Control and Prevention, National Center for Health Statistics, CDC WONDER Online Database, released June 2017. Data are from the Compressed Mortality File 1999–2016 Series 20 No. 2U, 2016, as compiled from data provided by the 57 vital statistics jurisdictions through the Vital Statistics Cooperative Program. Last accessed at http://wonder.cdc.gov/cmf-icd10.html on 31 Aug 2020 9:15:06 a.m. County Health Rankings 2018 data developed as a collaboration between the Robert Wood Johnson Foundation and the University of Wisconsin Population Health Institute were obtained from https://www.countyhealthrankings.org/explore-health-rankings/rankings-data-documentation (last access: 31 March 2020)^69^. Observed air quality data is available from the EPA Air Quality System (AQS) available at https://www.epa.gov/aqs (last access: 18 October 2021)^73,74^. Source data for data generated in this work are provided with this paper. Source data are provided with this paper.

## Source data

Source Data

## Code availability

The analysis code for post-processing CMAQ predictions, aggregating CMAQ to annual-average component values, aligning data by county, and performing the statistical analyses have been deposited at 10.5281/zenodo.5713903^74^. CMAQ v5.3.1 is available at 10.5281/zenodo.3585898^28^.
