# Supplementary material for: Secondary organic aerosol association with cardiorespiratory disease mortality in the United States
Source: Nat Commun. 2021 Dec 16;12:7215. doi: 10.1038/s41467-021-27484-1 (PMC8677800; doi:10.1038/s41467-021-27484-1)
Supplement: Supplementary file 1 — Supplementary Information [file 41467_2021_27484_MOESM1_ESM.pdf]

Supplementary Information for:

Secondary organic aerosol association with cardiorespiratory disease mortality in the United States

Havala O. T. Pye,[1*] Cavin K. Ward-Caviness,[1] Ben N. Murphy,[1] K. Wyat Appel,[1]

Karl M. Seltzer[2]

[1]Office of Research and Development, US Environmental Protection Agency, 109 TW Alexander Dr, Research Triangle Park, NC 27711

[2]Oak Ridge Institute for Science and Education Postdoctoral Fellow in the Office of Research and Development, US Environmental Protection Agency, 109 TW Alexander Dr, Research Triangle Park, NC 27711

[*]Correspondence to: pye.havala@epa.gov

Total Pages: 24
Number of Tables: 10
Number of Figures: 6

Supplementary Note 1: Additional sensitivity tests in statistical models

A state specific indicator was also considered but not included in main text results since many confounders from CHR 2018 were created with a statistical model that included a state-level factor, including the state factor led to increased error in the total $PM_{2.5}$ fitted coefficient from 0.47 to 0.60 deaths per 100,000 people on a 1 µg m^{-3} basis, and SOA contributions are expected to vary on a state to state basis due to differences in precursor emissions from vegetation as well as combustion sources. The results containing the state factor are available in the supplemental data archive and include an "ST" label in the notes.

Joint exposure to O_3 and NO_2 was considered in a sensitivity analysis (Supplementary Figure 2) but increased the standard error for the $PM_{2.5}$ coefficient from 0.47 to 0.74 deaths per 100,000 people on a 1 µg m^{-3} basis (Supplementary Table 5). Since including O_3 and NO_2 in multiple regression resulted in less precise estimates of the $PM_{2.5}$ mortality effect and positive associations between $PM_{2.5}$ and death rates were independent of these gaseous pollutants, O_3 and NO_2 were generally excluded from statistical models.

Multicollinearity between confounders was examined in the single pollutant SOA model for the contiguous U.S. One confounder had variance inflation factor (VIF) larger than 10 (non-hispanic white fraction, VIF=11.6). Removing the confounder variable with VIF>10 resulted in a minor decrease in the magnitude of association between SOA and CR mortality rates (from 7.79 to 6.11 additional deaths per 100,000 in population for an IQR increase in concentration). SOA remained statistically significant in its association with CR mortality rates.

Supplementary Figures 4 and 5 include additional statistical model results for two subcomponents of SOA_{BVOC}: $SOA_{ISOPRENE}$ and $SOA_{TERPENE}$. The multipollutant model shown in Supplementary Figure 5 includes the entire $PM_{2.5}$ composition with refinement of SOA subcomponents into $SOA_{ISOPRENE}$, $SOA_{TERPENE}$, and SOA_{AVOC}. The MP model in Supplementary Figure 5 is the same as MP SOA in the main text except SOA_{BVOC} is replaced with $SOA_{ISOPRENE}$ and $SOA_{TERPENE}$.

Supplementary Table 1: Summary of $PM_{2.5}$ component, confounder, and outcome values for counties with complete data across the contiguous (US, n=2,708) and in the southeastern (SE, n=646) United States. Detailed descriptions of the components are available in Supplementary Table 2. Additional information for the outcome and confounders is available in Supplementary Table 3. Minimum, maximum, and standard deviation (Std Dev) are for the contiguous US. Values are reported with 3 significant figures except for $PM_{2.5}$ components which are rounded to the hundredths of a $\mu g\ m^{-3}$.

	**Units**	**Minimum**	**Maximum**	**Std Dev**	**US Mean**	**US IQR**	**SE Mean**	**SE IQR**
**$PM_{2.5}$ Components**								
Total	$\mu g\ m^{-3}$	2.06	14.30	1.89	6.50	2.68	7.84	1.14
OA	$\mu g\ m^{-3}$	0.68	5.93	1.10	2.86	1.63	4.03	0.96
Dust	$\mu g\ m^{-3}$	0.40	6.13	0.52	1.49	0.69	1.60	0.46
SO_4	$\mu g\ m^{-3}$	0.39	1.62	0.23	0.93	0.29	1.03	0.14
NH_4NO_3	$\mu g\ m^{-3}$	0.13	2.25	0.39	0.76	0.46	0.63	0.31
Soot	$\mu g\ m^{-3}$	0.06	1.52	0.13	0.27	0.13	0.32	0.09
Sea spray	$\mu g\ m^{-3}$	0.07	1.16	0.12	0.18	0.09	0.23	0.12
**$PM_{2.5}$ OA Subcomponents**								
OC	$\mu gC\ m^{-3}$	0.36	3.95	0.63	1.58	0.92	2.24	0.60
POA	$\mu g\ m^{-3}$	0.16	4.31	0.27	0.66	0.27	0.77	0.22
SOA	$\mu g\ m^{-3}$	0.53	4.60	0.94	2.20	1.44	3.26	0.82
SOA_{AVOC}	$\mu g\ m^{-3}$	0.23	2.89	0.39	1.14	0.54	1.43	0.21
SOA_{BVOC}	$\mu g\ m^{-3}$	0.26	3.31	0.66	1.06	0.91	1.83	0.72
$SOA_{TERPENE}$	$\mu g\ m^{-3}$	0.13	2.40	0.46	0.59	0.51	1.12	0.65
$SOA_{ISOPRENE}$	$\mu g\ m^{-3}$	0.11	1.07	0.23	0.47	0.41	0.71	0.18
**Other pollutants**								
NO_2	ppb	0.152	19.6	1.55	2.18	1.33	2.14	0.985
O_3	ppb	19.5	41.6	2.50	30.8	2.36	30.1	2.69
**Environmental Variables**								
Relative Humidity	%	30.0	82.2	7.39	68.9	6.55	70.3	4.55
Temperature	K	274	298	4.25	287	6.53	291	3.72
Presence of drinking water violation	1 or 0	0	1	0.498	0.450	1	0.282	1
**Health Outcome**								
Cardiorespiratory deaths	Deaths per 100,000	95.5	779	80.7	330	103	374	111

	Units	Minimum	Maximum	Std Dev	US Mean	US IQR	SE Mean	SE IQR
**Behavioral, Social, and Economic Variables**								
Smoking adults	Fraction of adult population	0.0674	0.332	0.0347	0.178	0.0502	0.203	0.0374
Obese adults	Fraction of adult population	0.128	0.478	0.0448	0.314	0.054	0.333	0.048
Physically inactive adults	Fraction of adult population	0.098	0.444	0.0518	0.267	0.068	0.295	0.0587
Excessive drinking	Fraction of adult population	0.0927	0.294	0.0324	0.175	0.0454	0.151	0.0286
Number physicians	per 100,000	0	0.00453	0.000327	0.000556	0.000383	0.00049	0.000316
Diabetes patients	Fraction of adult population	0.227	0.977	0.0587	0.848	0.0572	0.855	0.0439
Adults with post-secondary education	Fraction of adult population	0.193	0.897	0.113	0.573	0.16	0.522	0.143
Unemployed	Fraction of adult population	0.0175	0.235	0.0177	0.0524	0.0205	0.0593	0.0177
Income inequality index	Ratio	2.97	8.93	0.709	4.52	0.841	4.87	0.9
Social associations	per 10,000	0.944	68.3	6.3	13.5	6.75	11.3	5.12
Violent crime offenses	per 100,000	0	1720	189	251	211	312	272
Housing problems	Fraction of households	0.0269	0.391	0.0419	0.145	0.0503	0.158	0.0434
Limited health food access	Fraction of population	0	0.593	0.0648	0.0769	0.0644	0.0694	0.0688
Uninsured adults	Fraction of population	0.0262	0.434	0.0613	0.14	0.0896	0.164	0.0557
Other health providers	per 100,000	0	0.0134	0.000492	0.000707	0.000467	0.000735	0.000475
Household income	Dollars	24200	135000	12900	49900	14200	43800	11400
**Demographic Variables**								
Population <18 years of age	Fraction of population	0.0707	0.366	0.0326	0.224	0.0369	0.22	0.034
Population ≥65 years of age	Fraction of population	0.0463	0.563	0.0442	0.183	0.0517	0.179	0.0421
American Indian and Alaskan Native	Fraction of population	0.000933	0.829	0.0575	0.019	0.00897	0.00737	0.00298
Asian	Fraction of population	0	0.365	0.025	0.0149	0.00962	0.0112	0.0088
Pacific Islander	Fraction of population	0	0.0277	0.00171	0.00106	0.000814	0.000943	0.000816
Hispanic	Fraction of population	0.00502	0.963	0.135	0.0942	0.0755	0.0562	0.0429
Non-hispanic White	Fraction of population	0.0281	0.98	0.192	0.771	0.266	0.717	0.316
Females	Fraction of population	0.278	0.565	0.0206	0.500	0.0148	0.506	0.0132
Rural population	Fraction of population	0	1	0.300	0.553	0.470	0.590	0.440
**Other**								
Population	People	669	10100000	348000	114000	64800	96400	65200

Supplementary Table 2: Aggregated CMAQ $PM_{2.5}$ components. CMAQ-predicted size distributions and the fraction of the Aitken, accumulation, and coarse modes (PM25AT, PM25AC, and PM25CO respectively) below 2.5 µm in diameter were used to calculate the mass in $PM_{2.5}$. [1] denotes the species is from the ACONC file. [2] denotes the quantity is from the APMDIAG file. See https://github.com/USEPA/CMAQ for CMAQ documentation.

**Component Name**	**Description**	**Subcomponent Species**	**CMAQ Internal Species**
OA	all primary and secondary organic aerosol	SOA + POA	(ALVPO1I[1]+ASVPO1I[1]+ASVPO2I[1]+APOCI[1]+APNCOMI[1] +ALVOO1I[1]+ALVOO2I[1]+ASVOO1I[1]+SVOO2I[1])*PM25AT[2] +(ALVPO1J[1]+ASVPO1J[1]+ASVPO2J[1]+APOCJ[1]+ASVPO3J[1]+AIVPO1J[1] +APNCOMJ[1]+AISO1J[1]+AISO2J[1]+AISO3J[1] +AMT1J[1]+AMT2J[1]+AMT3J[1]+AMT4J[1]+AMT5J[1]+AMT6J[1] +AMTNO3J[1]+AMTHYDJ[1]+AGLYJ[1]+ASQTJ[1] +AORGCJ[1]+AOLGBJ[1]+AOLGAJ[1] +ALVOO1J[1]+ALVOO2J[1]+ASVOO1J[1]+ASVOO2J[1]+ASVOO3J[1] +APCSOJ[1]+AAVB1J[1]+AAVB2J[1]+AAVB3J[1]+AAVB4J[1])*PM25AC[2]
Sea spray	Chloride, sodium, and magnesium	Cl + Na + Mg	ACLI[1]*PM25AT[2]+ACLJ[1]*PM25AC[2]+ACLK[1]*PM25CO[2]+ ANAI[1]*PM25AT[2]+ANAJ[1]*PM25AC[2] +(0.8373*ASEACAT[1]+0.0626*ASOIL[1]+0.0023*ACORS[1])*PM25CO[2]+ AMGJ[1]*PM25AC[2]+(0.0997*ASEACAT[1]+0.0170*ASOIL[1]+0.0032*ACORS[1])*PM25CO[2]
SO_4	total particulate sulfate (sulfate and bisulfate forms)	sulfate	ASO4I[1]*PM25AT[2]+ASO4J[1]*PM25AC[2]+ASO4K[1]*PM25CO[2]
Dust	Species associated with dust (e.g. Ca, Si) and other $PM_{2.5}$ not otherwise speciated	Ca + Fe + Si + Ti + Mn + Al + other unspeciated mass (UNSPCRS+OTHR)	ACAJ[1]*PM25AC[2]+(0.0320*ASEACAT[1]+0.0838*ASOIL[1]+0.0562*ACORS[1])*PM25CO[2]+ AOTHRI[1]*PM25AT[2]+AOTHRJ[1]*PM25AC[2]+ AFEJ[1]*PM25AC[2]+ ASIJ[1]*PM25AC[2]+ ATIJ[1]*PM25AC[2]+ AMNJ[1]*PM25AC[2]+ AALJ[1]*PM25AC[2]+ (ASOIL[1]+ACORS[1]+ASEACAT[1] -(0.8373*ASEACAT[1]+0.0626*ASOIL[1]+0.0023*ACORS[1]) -(0.0997*ASEACAT[1]+0.0170*ASOIL[1]+0.0032*ACORS[1]) -(0.0310*ASEACAT[1]+0.0242*ASOIL[1]+0.0176*ACORS[1]) -(0.0320*ASEACAT[1]+0.0838*ASOIL[1]+0.0562*ACORS[1])*PM25CO[2]
NH_4NO_3	Ammonium and nitrate	ammonium (NH_4) and nitrate (NO_3)	ANH4I[1]*PM25AT[2]+ANH4J[1]*PM25AC[2]+ANH4K[1]*PM25CO[2]+ ANO3I[1]*PM25AT[2]+ANO3J[1]*PM25AC[2]+ANO3K[1]*PM25CO[2]
Soot	Elemental carbon (EC) and potassium (K)	EC + K	AECI[1]*PM25AT[2]+AECJ[1]*PM25AC[2]+ AKJ[1]*PM25AC[2]+(0.0310*ASEACAT[1]+0.0242*ASOIL[1]+0.0176*ACORS[1])*PM25CO[2]

Component Name	Description	Subcomponent Species	CMAQ Internal Species
SOA	Secondary organic aerosol (formed in atmosphere)	all SOA = SOA_{BVOC} + SOA_{AVOC}	(ALVOO1I[1]+ALVOO2I[1]+ASVOO1I[1]+ASVOO2I[1])*PM25AT[2] +(AISO1J[1]+AISO2J[1]+AISO3J[1] +AMT1J[1]+AMT2J[1]+AMT3J[1]+AMT4J[1]+AMT5J[1]+AMT6J[1] +AMTNO3J[1]+AMTHYDJ[1]+AGLYJ[1]+ASQTJ[1]+AORGCJ[1]+AOLGBJ[1]+AOLGAJ[1] +ALVOO1J[1]+ALVOO2J[1]+ASVOO1J[1]+ASVOO2J[1]+ASVOO3J[1] +APCSOJ[1]+AAVB1J[1]+AAVB2J[1]+AAVB3J[1]+AAVB4J[1])*PM25AC[2]
POA	Primary organic aerosol (emitted)	POA	(ALVPO1I[1]+ASVPO1I[1]+ASVPO2I[1]+APOCI[1]+APNCOMI[1])*PM25AT[2] +(ALVPO1J[1]+ASVPO1J[1]+ASVPO2J[1]+APOCJ[1]+ASVPO3J[1]+AIVPO1J[1]+APNCOMJ[1])* PM25AC[2]
$SOA_{TERPENE}$	Monoterpene (C_{10}) and sesquiterpene (C_{15}) SOA	sesquiterpene (SQT), monoterpene nitrate + hydrolysis product (MTN), and monoterpene photooxidation (MT) SOA	ASQTJ[1]*PM25AC[2]+ (AMTNO3J[1]+AMTHYDJ[1])*PM25AC[2]+ (AMT1J[1]+AMT2J[1]+AMT3J[1]+AMT4J[1]+AMT5J[1]+AMT6J[1])*PM25AC[2]
$SOA_{ISOPRENE}$	Isoprene and correlated SOA including that from glyoxal, methylglyoxal, and biogenic oligomers	glyoxal+methylglyoxal SOA from aqueous uptake (GLYSOA), semivolatile isoprene SOA (ISOP), acid-catalyzed isoprene-epoxydiol SOA (IEPOX), and biogenic oligomers (OLGB)	(AORGCJ[1]+AGLYJ[1])*PM25AC[2]+ AOLGBJ[1]*PM25AC[2]+ (AISO1J[1]+AISO2J[1])*PM25AC[2]+ AISO3J[1]*PM25AC[2]
SOA_{BVOC}	SOA from VOCs that are predominantly biogenic in origin	$SOA_{TERPENE}$ + $SOA_{ISOPRENE}$	see above
SOA_{AVOC}	anthropogenic-VOC derived SOA	SOA from benzene, toluene, xylene, PAHs, long-chain alkanes, and other anthropogenic VOC sources	(AAVB1J[1]+AAVB2J[1]+AAVB3J[1]+AAVB4J[1]+AOLGAJ[1])*PM25AC[2] +(ALVOO1I[1]+ALVOO2I[1]+ASVOO1I[1]+ASVOO2I[1])*PM25AT[2] +(ALVOO1J[1]+ALVOO2J[1]+ASVOO1J[1]+ASVOO2J[1]+ASVOO3J[1]+APCSOJ[1])*PM25AC[2]

Supplementary Table 3: Confounders and other variables (variable names appear in supporting files available via data.gov) considered in statistical models. O_3 and NO_2 were only considered in some models.

**Model Variable**	**Confounder Description**	**Source**	**Original Data Source**	**Data Year**	**Units**
Adult.smoking.raw.value.2018uw	Adults that reported currently smoking every day or most days and have smoked at least 100 cigarettes	County Health Rankings 2018[a]	Behavioral Risk Factor Surveillance System (BRFSS)	2016	Fraction of Adults
Adult.obesity.raw.value.2018uw	Adults 20 and older with BMI >= 30 kg/m2 based on reported height and weight	County Health Rankings 2018	CDC Diabetes Interactive Atlas/BRFSS	2014	Fraction of Adults
Physical.inactivity.raw.value.2018uw	Adults 20 and older that report no leisure-time physical activity in past month	County Health Rankings 2018	CDC Diabetes Interactive Atlas	2014	Fraction of Adults
Excessive.drinking.raw.value.2018uw	Adults that report excessive drinking in last 30 days	County Health Rankings 2018	BRFSS	2016	Fraction of Adults
Primary.care.physicians.raw.value.2018uw	Number of primary care physicians (PCP) in patient care per 100,000 in population	County Health Rankings 2018	Area Health Resource File/American Medical Association	2015	Number physicians per 100,000 people
Diabetes.monitoring.raw.value.2018uw	Diabetic fee-for-service Medicare patients age 65-75 whose blood sugar control was monitored in the past year using their HbA1C levels	County Health Rankings 2018	Dartmouth Atlas of Health Care	2014	Fraction of Medicare Patients
Some.college.raw.value.2018uw	Adults age 25-44 with some post-secondary education	County Health Rankings 2018	American Community Survey	2012-2016	Fraction of Adults 25-44
Unemployment.raw.value.2018uw	People ages 16+ unemployed and looking for work	County Health Rankings 2018	Bureau of Labor Statistics	2016	Fraction
Income.inequality.raw.value.2018uw	Ratio of income at the 80th percentile to income at the 20th percentile	County Health Rankings 2018	American Community Survey	2012-2016	Ratio
Social.associations.raw.value.2018uw	Number of membership associations per 10,000 people	County Health Rankings 2018	County Business Patterns	2015	Number per 10,000
Violent.crime.raw.value.2018uw	Number of violent crime offenses per 100,000 people	County Health Rankings 2018	Uniform Crime Reporting – FBI	2012-2014	Number per 100,000

Model Variable	Confounder Description	Source	Original Data Source	Data Year	Units
Drinking.water.violations .raw.value.2018uw	County affected by a water violation: 1-Yes, 0-No	County Health Rankings 2018	Safe Drinking Water Information System	2016	1 or 0
Severe.housing.problems. raw.value.2018uw	Households with at least 1 of 4 housing problems: overcrowding, high housing costs, lack of kitchen, or lack of plumbing facilities	County Health Rankings 2018	Comprehensive Housing Affordability Strategy (CHAS) data	2010-2014	Fraction of households
Limited.access.to.healthy. foods.raw.value.2018uw	Population that is low-income and not close to a grocery store	County Health Rankings 2018	USDA Food Environment Atlas	2015	Fraction of population
Uninsured.adults.raw. value.2018uw	Adults under age 65 without health insurance	County Health Rankings 2018	Small Area Health Insurance Estimates	2015	Fraction of population age 18 to 64
Other.primary.care. providers.raw.value.2018 uw	Primary care providers that are not physicians (nurse practitioners, physician assistants, clinical nurse specialists, etc).	County Health Rankings 2018	Centers for Medicare & Medicaid Services (CMS), National Provider Identification file	2017	Number of providers per 100,000 people
Median.household. income.raw.value. 2018uw	Median income	County Health Rankings 2018	Small Area Income and Poverty Estimates	2016	Dollars
X..below.18.years.of.age. raw.value.2018uw	Population below 18 years of age	County Health Rankings 2018	Census Population Estimates Program	2016	Fraction of population
X..65.and.older.raw.value .2018uw	Population 65 years of age and older	County Health Rankings 2018	Census Population Estimates Program	2016	Fraction of population
X..American.Indian.and. Alaskan.Native.raw.value .2018uw	American Indian and Alaskan Native population	County Health Rankings 2018	Census Population Estimates Program	2016	Fraction of population
X..Asian.raw.value.2018 uw	Asian population	County Health Rankings 2018	Census Population Estimates Program	2016	Fraction of population
X..Native.Hawaiian. Other.Pacific.Islander. raw.value.2018uw	Native Hawaiian/Other Pacific Islander population	County Health Rankings 2018	Census Population Estimates Program	2016	Fraction of population

**Model Variable**	**Confounder Description**	**Source**	**Original Data Source**	**Data Year**	**Units**
X..Hispanic.raw.value. 2018uw	Hispanic population	County Health Rankings 2018	Census Population Estimates Program	2016	Fraction of population
X..Non.Hispanic.white. raw.value.2018uw	Non-Hispanic White population	County Health Rankings 2018	Census Population Estimates Program	2016	Fraction of population
X..Females.raw.value. 2018uw	Female population	County Health Rankings 2018	Census Population Estimates Program	2016	Fraction of population
X..Rural.raw.value. 2018uw	Population in rural area (census tract <2,500 people)	County Health Rankings 2018	Census Population Estimates Program	2010	Fraction of population
RH.percent.cmaq	Relative Humidity	CMAQ Modeling System	WRF v4.1.1 Prediction	2016	percent
SFC_TMP.K.cmaq	Surface Air Temperature	CMAQ Modeling System	WRF v4.1.1 Prediction	2016	K
Population.people.5yracs	Population by county	Census/5 yr American Community Survey (ACS)	Variable S0101_C01_001E from ACS Table S0101	2012-2016	People
O3.ppbV.cmaq	Ozone concentration	CMAQ Modeling System	CMAQ v5.3.1 Prediction	2016	ppbV
NO2.ppbV.cmaq	NO_2 concentration	CMAQ Modeling System	CMAQ v5.3.1 Prediction	2016	ppbV

[a]County Health Rankings 2018 data developed as a collaboration between the Robert Wood Johnson Foundation and the University of Wisconsin Population Health Institute were obtained from https://www.countyhealthrankings.org/explore-health-rankings/rankings-data-documentation. Full documentation, including a Data Dictionary, is available at the link. Date of last access: 31 March 2020.

Supplementary Table 4: Correlation (Pearson r) between final $PM_{2.5}$ aggregates (columns) and species (rows) predicted by CMAQ for the contiguous US. Red indicates r > 0.8.

	TOT	OA	SO_4	SEASPRAY	DUST	NH_4NO_3	SOOT	POA	SOA	SOA_{BVOC}	SOA_{AVOC}	$SOA_{ISOPRENE}$	$SOA_{TERPENE}$
**CL**	0.05	0.12	0.07	0.99	-0.15	-0.28	0.15	0.10	0.12	0.16	0.01	0.07	0.20
**EC**	0.77	0.63	0.52	0.12	0.58	0.55	0.99	0.78	0.52	0.30	0.74	0.37	0.24
**NA**	0.10	0.23	0.17	0.99	-0.15	-0.39	0.12	0.08	0.24	0.32	0.05	0.23	0.34
**MG**	0.27	0.30	0.26	0.95	0.10	-0.22	0.32	0.19	0.30	0.31	0.19	0.26	0.31
**K**	0.83	0.72	0.59	0.22	0.66	0.44	0.88	0.69	0.65	0.47	0.78	0.51	0.41
**CA**	0.68	0.36	0.56	-0.03	0.95	0.52	0.56	0.35	0.33	0.18	0.49	0.32	0.10
**NH_4**	0.63	0.31	0.65	-0.42	0.61	0.94	0.50	0.41	0.25	0.00	0.59	0.23	-0.12
**NO_3**	0.52	0.15	0.37	-0.29	0.58	0.99	0.55	0.40	0.06	-0.20	0.48	-0.06	-0.25
**OC**	0.88	1.00	0.63	0.18	0.43	0.21	0.68	0.74	0.97	0.88	0.84	0.86	0.83
**OM**	0.88	1.00	0.67	0.19	0.43	0.21	0.66	0.71	0.98	0.89	0.85	0.89	0.83
**OTHR**	0.83	0.56	0.67	-0.06	0.97	0.57	0.68	0.57	0.50	0.33	0.65	0.46	0.24
**FE**	0.65	0.27	0.57	-0.18	0.94	0.70	0.54	0.32	0.22	0.03	0.49	0.20	-0.06
**SI**	0.68	0.32	0.58	-0.15	0.94	0.67	0.59	0.34	0.28	0.09	0.52	0.25	0.00
**TI**	0.63	0.29	0.54	-0.19	0.83	0.68	0.54	0.36	0.24	0.06	0.47	0.22	-0.02
**MN**	0.65	0.28	0.57	-0.18	0.91	0.67	0.52	0.31	0.24	0.06	0.49	0.21	-0.02
**AL**	0.57	0.19	0.55	-0.25	0.91	0.66	0.41	0.22	0.16	-0.01	0.40	0.17	-0.10
**UNSPCRS**	0.50	0.16	0.38	-0.27	0.90	0.51	0.36	0.20	0.14	0.01	0.31	0.15	-0.06
**GLYSOA**	0.85	0.93	0.77	0.10	0.46	0.19	0.52	0.54	0.95	0.87	0.81	0.97	0.76
**OLGB**	0.66	0.81	0.70	0.25	0.29	-0.08	0.31	0.37	0.85	0.88	0.57	0.97	0.77
**ISOP**	0.68	0.88	0.54	0.07	0.28	-0.03	0.38	0.46	0.90	0.90	0.66	0.92	0.83
**IEPOX**	0.62	0.71	0.80	0.27	0.27	-0.02	0.29	0.38	0.73	0.73	0.51	0.91	0.59
**SQT**	0.52	0.76	0.36	0.23	0.14	-0.17	0.21	0.25	0.82	0.93	0.41	0.75	0.95
**MTN**	0.65	0.90	0.45	0.26	0.21	-0.13	0.41	0.44	0.94	0.97	0.62	0.85	0.95
**MT**	0.43	0.73	0.22	0.30	0.02	-0.28	0.17	0.25	0.79	0.92	0.35	0.67	0.97
**$SOA_{ISOPRENE}$**	0.75	0.89	0.74	0.18	0.36	0.03	0.41	0.47	0.92	0.90	0.69	1.00	0.79
**$SOA_{TERPENE}$**	0.55	0.83	0.35	0.28	0.12	-0.21	0.28	0.34	0.89	0.98	0.49	0.79	1.00
**TOT**	1.00	0.88	0.82	0.09	0.76	0.57	0.81	0.74	0.83	0.65	0.90	0.75	0.55
**OA**	0.88	1.00	0.67	0.19	0.43	0.21	0.66	0.71	0.98	0.89	0.85	0.89	0.83
**SO_4**	0.82	0.67	1.00	0.14	0.61	0.47	0.55	0.47	0.65	0.50	0.72	0.74	0.35
**SEASPRAY**	0.09	0.19	0.14	1.00	-0.13	-0.34	0.14	0.10	0.20	0.26	0.04	0.18	0.28
**DUST**	0.76	0.43	0.61	-0.13	1.00	0.61	0.61	0.45	0.38	0.21	0.56	0.36	0.12
**NH_4NO_3**	0.57	0.21	0.47	-0.34	0.61	1.00	0.54	0.41	0.12	-0.14	0.53	0.03	-0.21
**SOOT**	0.81	0.66	0.55	0.14	0.61	0.54	1.00	0.79	0.56	0.34	0.77	0.41	0.28
**POA**	0.74	0.71	0.47	0.10	0.45	0.41	0.79	1.00	0.55	0.40	0.65	0.47	0.34
**SOA**	0.83	0.98	0.65	0.20	0.38	0.12	0.56	0.55	1.00	0.94	0.82	0.92	0.89
**SOA_{BVOC}**	0.65	0.89	0.50	0.26	0.21	-0.14	0.34	0.40	0.94	1.00	0.58	0.90	0.98
**SOA_{AVOC}**	0.90	0.85	0.72	0.04	0.56	0.53	0.77	0.65	0.82	0.58	1.00	0.69	0.49

Supplementary Table 5: Multiple regression model results for SOA and $PM_{2.5}$ (full results available in statmodels.pm25models.cYYYYMMDD.csv). Results include regressed coefficients for 1 μg^{-1} m^3 of pollutant ($\beta_{1\mu g}$), standard error (SE), p-value, population-weighted concentration (Conc.), IQR of pollutant or pollutant residual, presence of O_3 and NO_2 in model, adjustment for $PM_{2.5}$, region, type of model, and regressed coefficient for IQR normalized concentration (β_{IQR}).

Pollutant	$\beta_{1\mu g}$	$\beta_{1\mu g}$ 95% CI		SE	p-val.	Conc.	IQR	O_3NO_2	PM-adj	Region	Model	β_{IQR}	β_{IQR} 95% CI	
	deaths 10^{-5} μg^{-1} m^3					$\mu g\ m^{-3}$	$\mu g\ m^{-3}$					deaths 10^{-5}		
PM25_SOA	5.4	2.9	7.9	1.3	3E-05	2.35	1.44	FALSE	FALSE	CONUS	SP PM25	7.8	4.2	11.4
PM25_SOA	5.4	1.9	9.0	1.8	3E-03	2.35	1.44	FALSE	TRUE	CONUS	SP-adj PM25	7.8	2.7	12.9
PM25_SOA	1.7	-1.4	4.7	1.6	3E-01	2.35	0.70	FALSE	FALSE	CONUS	R PM25	1.2	-1.0	3.3
PM25_SOA	8.9	6.0	11.9	1.5	4E-09	2.35	1.44	FALSE	FALSE	CONUS	MP OA	12.8	8.6	17.1
PM25_SOAAVOC	6.8	2.9	10.7	2.0	6E-04	1.37	0.54	FALSE	FALSE	CONUS	SP PM25	3.7	1.6	5.8
PM25_SOAAVOC	5.7	-0.5	11.8	3.1	7E-02	1.37	0.54	FALSE	TRUE	CONUS	SP-adj PM25	3.1	-0.3	6.4
PM25_SOAAVOC	5.5	-0.6	11.7	3.1	8E-02	1.37	0.19	FALSE	FALSE	CONUS	R PM25	1.1	-0.1	2.2
PM25_SOAAVOC	6.2	0.5	11.8	2.9	3E-02	1.37	0.54	FALSE	FALSE	CONUS	MP SOA	3.4	0.3	6.4
PM25_SOABVOC	6.9	2.7	11.0	2.1	1E-03	0.98	0.91	FALSE	FALSE	CONUS	SP PM25	6.2	2.5	10.0
PM25_SOABVOC	5.4	1.0	9.8	2.2	2E-02	0.98	0.91	FALSE	TRUE	CONUS	SP-adj PM25	4.9	1.0	8.9
PM25_SOABVOC	0.4	-3.1	4.0	1.8	8E-01	0.98	0.61	FALSE	FALSE	CONUS	R PM25	0.3	-1.9	2.4
PM25_SOABVOC	11.1	6.3	15.8	2.4	5E-06	0.98	0.91	FALSE	FALSE	CONUS	MP SOA	10.1	5.8	14.4
PM25_TOT	1.4	0.5	2.3	0.5	3E-03	7.42	2.68	FALSE	FALSE	CONUS	SP PM25	3.7	1.2	6.2
PM25_TOT	2.4	0.9	3.8	0.7	1E-03	7.42	2.68	TRUE	FALSE	CONUS	SP PM25 O3,NO2	6.3	2.4	10.2
PM25_SOA	24.9	15.2	34.7	5.0	7E-07	3.05	0.82	FALSE	FALSE	SoutheastUS	SP PM25 SEast	20.5	12.5	28.5
PM25_SOA	21.5	9.1	33.9	6.3	7E-04	3.05	0.82	FALSE	TRUE	SoutheastUS	SP-adj PM25 SEast	17.7	7.5	27.9
PM25_SOA	27.1	16.1	38.2	5.6	2E-06	3.05	0.82	FALSE	FALSE	SoutheastUS	MP OA SEast	22.3	13.2	31.4
PM25_SOA	10.8	-1.0	22.7	6.0	7E-02	3.05	0.52	FALSE	FALSE	SoutheastUS	R PM25 SEast	5.6	-0.5	11.7
PM25_SOAAVOC	-8.0	-38.3	22.3	15.4	6E-01	1.44	0.21	FALSE	FALSE	SoutheastUS	SP PM25 SEast	-1.7	-7.9	4.6
PM25_SOAAVOC	-69.1	-106.6	-31.6	19.1	3E-04	1.44	0.21	FALSE	TRUE	SoutheastUS	SP-adj PM25 SEast	-14.3	-22.1	-6.5
PM25_SOAAVOC	-53.7	-100.7	-6.7	23.9	3E-02	1.44	0.21	FALSE	FALSE	SoutheastUS	MP SOA SEast	-11.1	-20.9	-1.4
PM25_SOAAVOC	-80.0	-117.2	-42.9	18.9	3E-05	1.44	0.13	FALSE	FALSE	SoutheastUS	R PM25 SEast	-10.5	-15.4	-5.6
PM25_SOABVOC	28.1	18.0	38.2	5.2	7E-08	1.61	0.72	FALSE	FALSE	SoutheastUS	SP PM25 SEast	20.2	12.9	27.5
PM25_SOABVOC	24.1	12.8	35.4	5.7	3E-05	1.61	0.72	FALSE	TRUE	SoutheastUS	SP-adj PM25 SEast	17.3	9.2	25.4
PM25_SOABVOC	34.4	22.7	46.0	5.9	1E-08	1.61	0.72	FALSE	FALSE	SoutheastUS	MP SOA SEast	24.7	16.3	33.1
PM25_SOABVOC	16.4	5.4	27.5	5.6	4E-03	1.61	0.50	FALSE	FALSE	SoutheastUS	R PM25 SEast	8.2	2.7	13.7
PM25_TOT	9.1	4.4	13.8	2.4	2E-04	7.88	1.14	FALSE	FALSE	SoutheastUS	SP PM25 SEast	10.4	5.0	15.8

Supplementary Table 6: Results of single and multi-pollutant multiple regression models for inorganic species in Figure 3. Models are for the contiguous U.S. unless otherwise noted as the Southeast (SEast).

Pollutant	Model	β_{IQR}	β_{IQR} 95% CI Lower Bound	β_{IQR} 95% CI Upper Bound
		deaths 10^{-5}	deaths 10^{-5}	deaths 10^{-5}
PM25_SO4	SP PM25	4.5	1.8	7.2
PM25_SOA	SP PM25	7.8	4.2	11.4
PM25_SEASPRAY	SP PM25	0.7	-0.8	2.1
PM25_DUST	SP PM25	2.9	1.2	4.5
PM25_NH4NO3	SP PM25	3.3	1.4	5.1
PM25_SOOT	SP PM25	-2.3	-3.3	-1.2
PM25_SEASPRAY	MP	2.7	1.1	4.3
PM25_SOOT	MP	-8.1	-9.7	-6.5
PM25_DUST	MP	5.1	2.6	7.6
PM25_NH4NO3	MP	2.6	-0.3	5.5
PM25_SO4	MP	3.9	-0.3	8.1
PM25_SEASPRAY	MP OA	2.7	1.1	4.3
PM25_SOOT	MP OA	-6.9	-9.3	-4.6
PM25_DUST	MP OA	4.8	2.3	7.3
PM25_NH4NO3	MP OA	2.6	-0.3	5.5
PM25_SO4	MP OA	4.0	-0.3	8.2
PM25_SOA	MP OA	12.8	8.6	17.1
PM25_SO4	SP PM25 SEast	2.4	-3.4	8.3
PM25_SOA	SP PM25 SEast	20.5	12.5	28.5
PM25_SEASPRAY	SP PM25 SEast	-5.1	-14.0	3.9
PM25_DUST	SP PM25 SEast	4.9	0.2	9.7
PM25_NH4NO3	SP PM25 SEast	8.0	-1.1	17.0
PM25_SOOT	SP PM25 SEast	0.5	-3.3	4.4
PM25_SEASPRAY	MP SEast	7.0	-3.3	17.4
PM25_SOOT	MP SEast	-6.9	-12.5	-1.4
PM25_DUST	MP SEast	4.6	-2.5	11.7
PM25_NH4NO3	MP SEast	10.7	0.6	20.8
PM25_SO4	MP SEast	-0.6	-10.1	8.9
PM25_SEASPRAY	MP OA SEast	7.6	-2.8	17.9
PM25_SOOT	MP OA SEast	-4.2	-10.6	2.3
PM25_DUST	MP OA SEast	4.9	-2.2	12.0
PM25_NH4NO3	MP OA SEast	9.1	-1.1	19.4
PM25_SO4	MP OA SEast	-2.0	-11.6	7.7
PM25_SOA	MP OA SEast	22.3	13.2	31.4

Supplementary Table 7: Results of single pollutant multiple regression models for OC (modeled by CMAQ and observed at AQS sites) at counties with AQS sites only. All OC in µgC m^{-3}. Models are for the contiguous U.S. and do not include exposure to O_3 or NO_2 or $PM_{2.5}$ adjustment. Variables are the same as those in Supplementary Table 5.

**Pollutant**	$\beta_{1\mu g}$	$\beta_{1\mu g}$ **95% CI**		**SE**	**p-val.**	**Conc.**	**IQR**	**Model**	β_{IQR}	β_{IQR} **95% CI**	
	deaths 10^{-5} µgC^{-1} m^3					µgC m^{-3}	µgC m^{-3}		deaths 10^{-5}		
PM25_OCmodel	-1.3	-12	9.3	5.4	0.81	1.7	1.2	SP	-1.6	-14	11
PM25_OCobserved	4.1	1.1	7.0	1.5	0.007	2.0	1.2	SP	4.9	1.3	8.4

Supplementary Table 8: CMAQ v5.3.1 simulation configuration (as in the work of Appel, et al. [1] CMAQ531_WRF411_M3Dry_BiDi).

**Option**	**Configuration**
Chemical mechanism	cb6r3
Aerosol module	aero7
POA volatility	semivolatile
Anthropogenic SOA (pcSOA)	pcSOA included from anthropogenic sources other than wood burning
Meteorology	WRF v4.1.1 processed with MCIP v5.0
Deposition	M3Dry
Boundary conditions	Hemispheric CMAQ v5.3.1 with CB6R3M_AE7_KMTBR and WRF3.8
Bi-directional ammonia emission	Yes
Emission platform	2016v1 (fh) based on 2014 NEI

Supplementary Table 9: Comparison of AQS-observed and CMAQ model-predicted $PM_{2.5}$ and its components across the US for 2016. Green shading indicates the metric meets the goal level for that component and/or concentration level. Blue shading indicates the metric meets the criteria performance metric for that component. NMB goal and criteria levels are component specific as specified in the work of Emery, et al. [2] NMB criteria are not available for dust or sea spray components. The MFB and MFE goals are a function of concentration as specified in the work of Boylan and Russell [3].

Component	Median Observed [μg m^{-3}]	Median Predicted [μg m^{-3}]	IQR Observed [μg m^{-3}]	IQR Predicted [μg m^{-3}]	NMB[e]	MFB[f]	MFE[g]
$PM_{2.5}$	6.63	6.64	5.45	5.90	2%	2%	42%
OC[a]	0.93	1.01	1.21	1.48	7%	10%	50%
Dust[b]	0.19	0.27	0.28	0.39	22%	32%	77%
SO_4	0.59	0.73	0.75	0.62	8%	23%	46%
NH_4NO_3	0.47	0.33	0.73	0.58	-20%	-21%	59%
Soot[c]	0.21	0.21	0.38	0.42	8%	10%	51%
Sea spray[d]	0.05	0.08	0.11	0.11	-5%	48%	82%

[a]Units of OC are μgC m^{-3}

[b]Dust is evaluated using the sum of Ca + Fe + Si + Ti + Mn + Al

[c]Soot is evaluated as EC + K

[d]Sea spray is evaluated as Na + Cl + Mg

[e]Normalized mean bias (NMB): $\frac{\sum_{i=1}^{N} M_i - O_i}{\sum_{i=1}^{N} O_i} \times 100\%$, where M_i is the model prediction and O_i is the observation and N is the total number of daily observations.

[f]Mean fractional bias (MFB): $\frac{2}{N}\sum_{i=1}^{N} \frac{M_i - O_i}{M_i + O_i} \times 100\%$

[g]Mean fractional error (MFE): $\frac{2}{N}\sum_{i=1}^{N} \frac{|M_i - O_i|}{M_i + O_i} \times 100\%$

Supplementary Table 10: CMAQ organic aerosol evaluation with non-routine and/or source resolved measurements for the contiguous US. Italics indicate systems and algorithms that motivated updates to CMAQ and were replaced or modified in later model versions. See table footnote for abbreviations.

OA System	CMAQ version	Model Algorithm Basis	Evaluation Study	Measurements	Evaluation Results
Isoprene SOA	*v5.0.1 with Isoprene SOA as in v5.1*	*Pye, et al. [4]*	*Pye, et al. [4]*	*Speciated isoprene SOA (2-methyltetrols and 2-methylglyceric acid) from filter-based analysis across the US*	*New algorithms improved CMAQ predictions of isoprene SOA tracers; Sensitivity to rate constants and Henry's law parameters identified*
Isoprene SOA	*v5.0.1 with Isoprene SOA as in v5.1*	*Pye, et al. [4]*	*Karambelas, et al. [5]*	*IEPOX OA PMF Factor from ACSM operated at Jefferson Street site in Atlanta during summer 2011*	*Strong temporal correspondence between CMAQ-predicted and PMF-observed IEPOX OA; Model IEPOX OA magnitude low*
Isoprene SOA	*v5.1 in box model*	*box model based on Pye, et al. [4] with updates as in Budisulistiorini, et al. [6]*	*Budisulistiorini, et al. [6]*	*Isoprene-derived organosulfates and 2-methyltetrols at SOAS-LRK*	*Correlation (r^2) between box model-predicted and observed tracers about 0.6.*
Isoprene SOA	v5.2	Pye, et al. [4] framework with Pye, et al. [7] parameter updates	Pye, et al. [7]	Isoprene-OA or similar AMS/ACSM factor at SOAS-CTR and SOAS-LRK; 2-methytetrols at SOAS-CTR	Isoprene-OA vs. sulfate trend (slope and correlation) similar in model and observations; Isoprene-OA NMB 10% at CTR and -39% at LRK; 2-methyltetrol NMB -22% at CTR
Multiple biogenic systems	v5.1 with SOA+POA updates as in v5.2	Pye, et al. [8]; Pye, et al. [7]; Murphy, et al. [9]	Liu, et al. [10]	AMS, ACSM, and FTIR PMF factors including multiple types of biogenic SOA at SOAS-CTR and SOAS-LRK	Qualitative consistency between CMAQ and observations in terms of regional abundance of isoprene vs monoterpene SOA and spatial variability including NO_x-driven enhancements of SOA at SOAS-CTR
Monoterpene (MT) SOA	*v5.1*	*Carlton, et al. [11]*	*Zhang, et al. [12]*	*~334 molecular formulas from GC + additional from FIGAERO-CIMS attributed to monoterpene oxidation at SOAS-CTR (approx. 55% of OOA)*	*Monoterpene SOA significantly underestimated in CMAQ v5.1*
LO-OOA (later associated with monoterpene SOA)	*v5.1 with traditional SOA updates as in v5.2*	*Pye, et al. [7]*	*Pye, et al. [7]*	*LO-OOA and OM/OC from SOAS-CTR[2]*	*LO-OOA underestimated in CMAQ (by ~50%); OM/OC reasonable*

OA System	CMAQ version	Model Algorithm Basis	Evaluation Study	Measurements	Evaluation Results
MT SOA	*v5.2*	*Carlton, et al.* [11]	*Pye, et al.* [13]	*a-pinene low-NO_x laboratory experiment from SOAFFEE*	*CMAQ v5.2 predicted SOA yield (7%) much lower than observed (12%) in the experiment.*
Monoterpene organic nitrate-derived SOA (subset of MT SOA)	v5.1-beta	Pye, et al. [8]	Pye, et al. [8]	SOAS CTR LO-OOA; AMS particulate organic nitrate (pON)	60% of LO-OOA captured by organic nitrate SOA pathway; model-predicted pON abundance within observation uncertainty
MT SOA (and others)	v5.3	Pye, et al. [8]; Xu, et al. [14]; semivolatile POA as in Murphy, et al. [9]	Lee, et al. [15]	FIGAERO-CIMS molecular formulas from SOAS-CTR attributed to precursor system using SOAFFEE laboratory data	Both observations (year 2013) and model predictions (year 2016) qualitatively consistent in terms of monoterpene SOA dominance (~60% of total OA); limited isoprene SOA and other SOA sources similar in observations and model predictions
MT SOA	v5.2 updated with MTSOA as in v5.3	Pye, et al. [8]; Xu, et al. [14]	Xu, et al. [14]	AMS+ACSM LO-OOA as a surrogate for monoterpene and sesquiterpene SOA; observations around the southeast US at different times of year	Updated CMAQ SOA (as in v5.3) consistent in terms of magnitude and diurnal variation with MT SOA as indicated by LO-OOA
Anthropogenic SOA and semivolatile POA	v5.2	Murphy, et al. [9]	Murphy, et al. [9]	CalNex, CARES, and SOAS-CTR (as well as IMPROVE, CSN, and SEARCH network data) including AMS PMF factors	CMAQ updated with semivolatile POA+empirical anthropogenic SOA (as in v5.2-5.3) showed improved total OA mass in terms of correlation and bias; HOA, OOA, ratio of HOA:OOA and diurnal variation in OA improved significantly
Anthropogenic SOA	v5.3	Murphy, et al. [9]		CalNex Pasadena AMS OOA (normalization and/or background corrections applied)	Anthropogenic SOA implementation in CMAQ represents both combustion and non-combustion (e.g. volatile chemical product) SOA reflecting current state-of-science on anthropogenic SOA sources

Abbreviations

ACSM: Aerosol Chemical Speciation Monitor

AMS: Aerosol Mass Spectrometer, more specifically the High-Resolution Time-of Flight Aerosol Mass Spectrometer (HR-ToF-AMS)

CalNex: California Research at the Nexus of Air Quality and Climate Change field campaign with supersite in Pasadena and Bakersfield, California, USA during May-June 2010

CARES: Carbonaceous Aerosols and Radiative Effects Study field campaign in northern California, June 2010
FIGAERO-CIMS: Filter Inlet for Gases and Aerosols on Chemical Ionization Spectrometer
FTIR: Fourier-transform infrared spectroscopy
HOA: Hydrocarbon-like organic aerosol
IEPOX-OA: SOA from isoprene epoxydiols
LV-OOA: Low Volatility OOA
LO-OOA: Less-Oxidized OOA
NMB: Normalized mean bias
OOA: Oxygenated organic aerosol, usually estimated via PMF
PMF: Positive matrix factorization
SOAFFEE: Secondary Organic Aerosol from Forest Emissions Experiment
SOAS: Southern Oxidant and Aerosol Study field campaign in the southeastern U.S. during June 2013
SOAS-CTR: Centreville, Alabama, USA site during SOAS 2013
SOAS-LRK: Look Rock, Tennessee, USA site during SOAS 2013
SV-OOA: Semi-Volatile OOA

Supplementary Figure 1: 2016 annual-average county-level concentration of aerosol species predicted by CMAQv5.3.1. Dust, soot, and POA color bar scales are capped at the 99th percentile of predicted values.

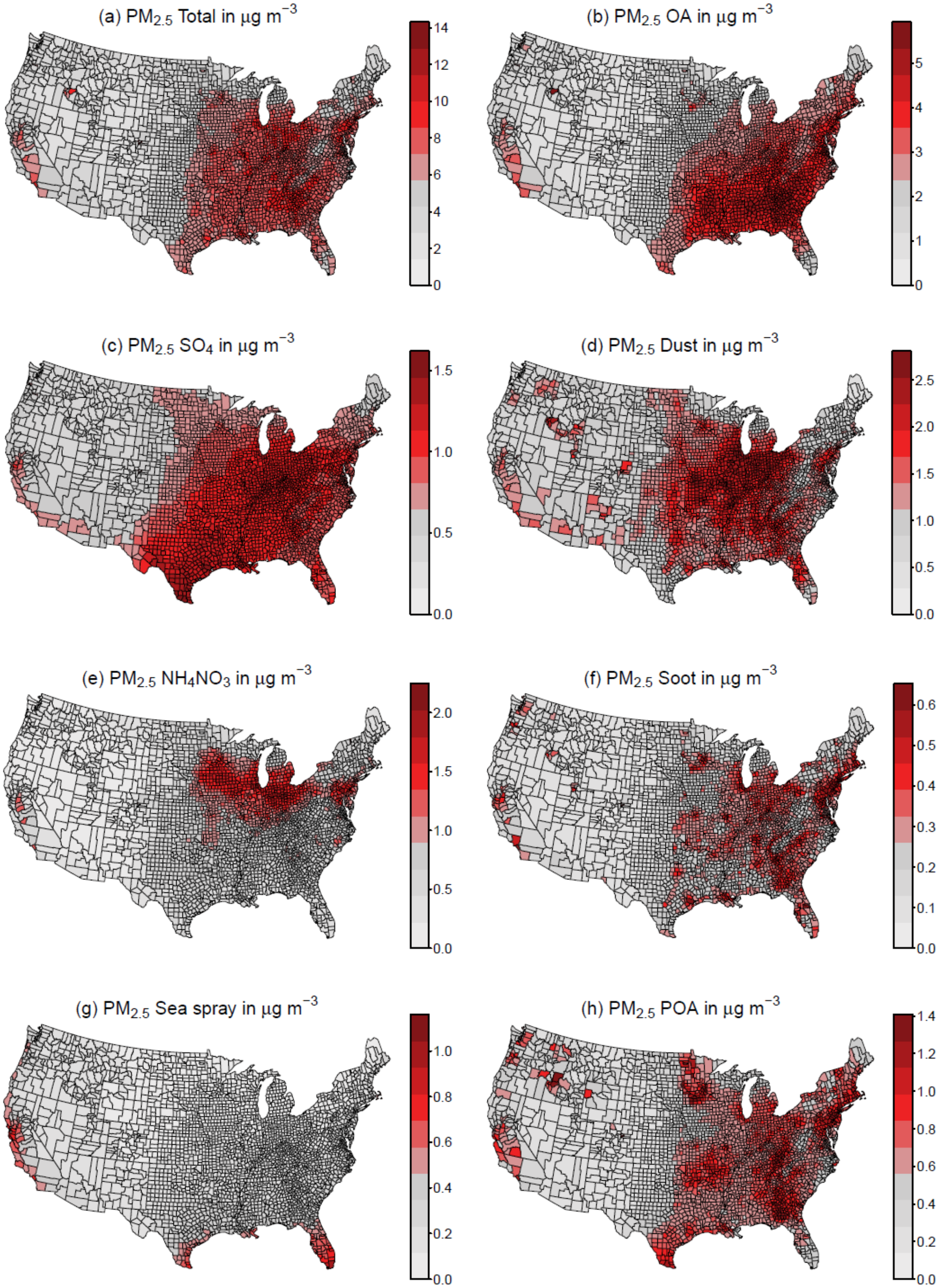

Supplementary Figure 1: continued

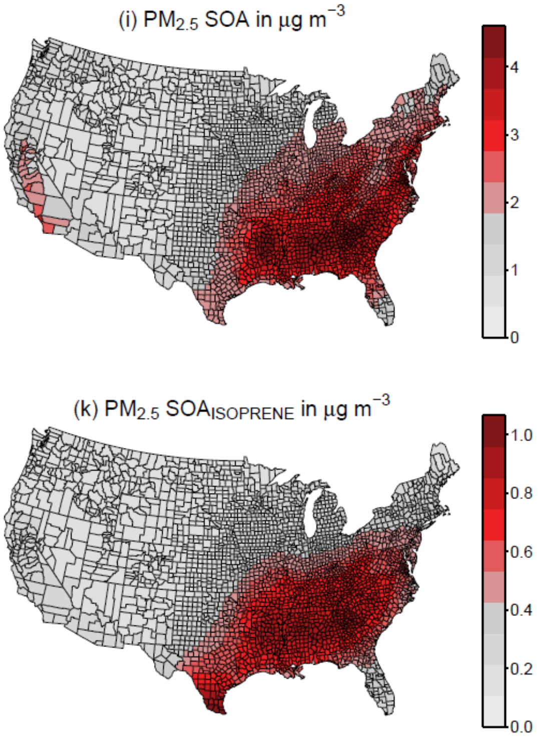

Supplementary Figure 2: Primary statistical analyses and associated sensitivity tests.

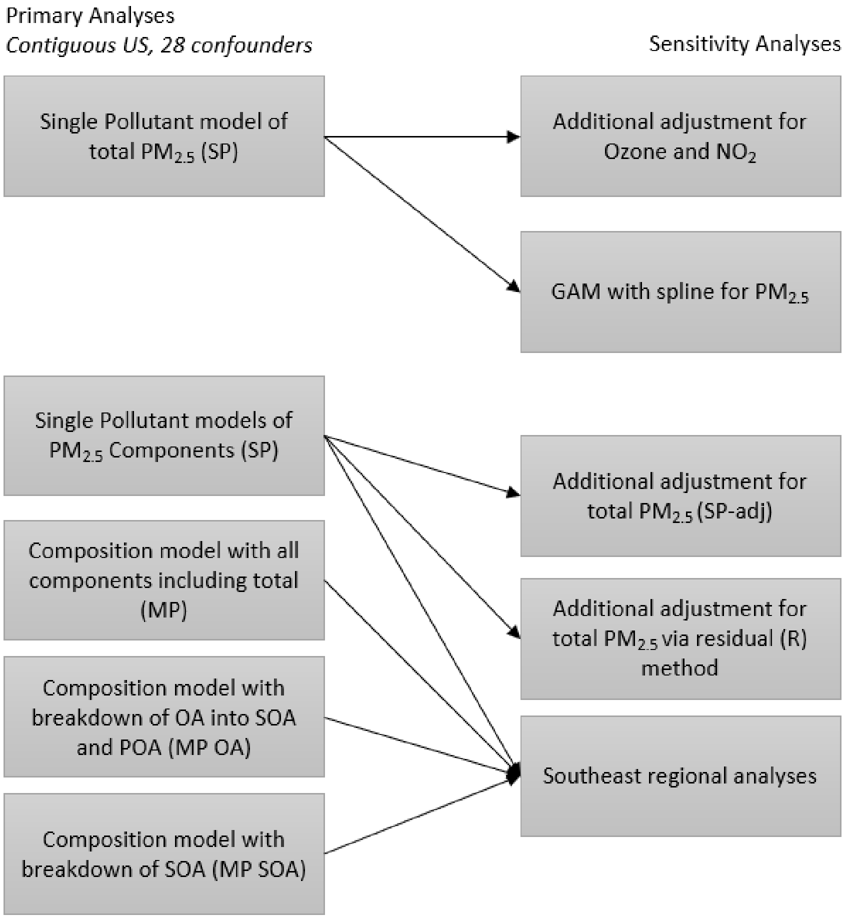

Supplementary Figure 3: Relationship between outcome (cardiorespiratory death rate) and $PM_{2.5}$ (a) total (PM25_TOT) and (b) SOA (PM25_SOA) concentration determined via thin plate spline in GAM framework. The basis dimension on the pollutant spline is set to 5 (k parameter in mgcv spline) in each case. Standard confounders are included. Shading represents two standard error bounds around the fit. $PM_{2.5}$ concentrations are shown on the horizontal axis in black vertical lines. Panel (a) for total $PM_{2.5}$ is limited to concentrations between the 5th and 95th percentile (2.9 and 9.0 μg m^{-3}) of the distribution.

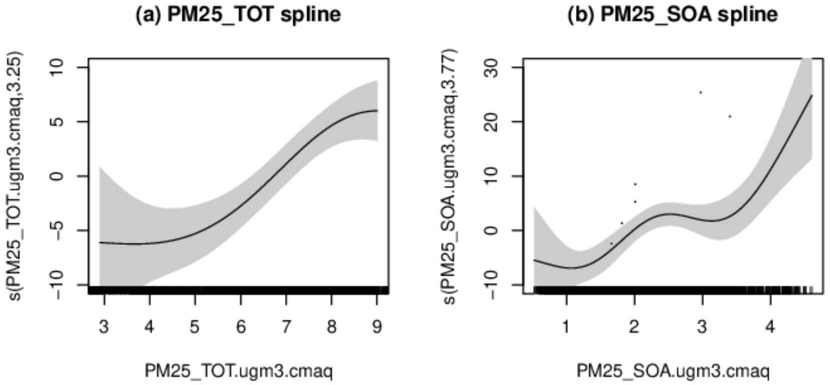

Supplementary Figure 4: Regressed coefficients (species normalized to IQR of pollutant or residual, open symbol) for the contiguous US (n=2,708) and southeastern (SE) US (n=646) using the 28 default confounders in (a, c) single pollutant adjusted for total $PM_{2.5}$ (SP-adj) and (b, d) residual (R) forms of multiple regression. Coefficients are ranked by the magnitude of the regressed coefficient. Whiskers span the 95% confidence interval.

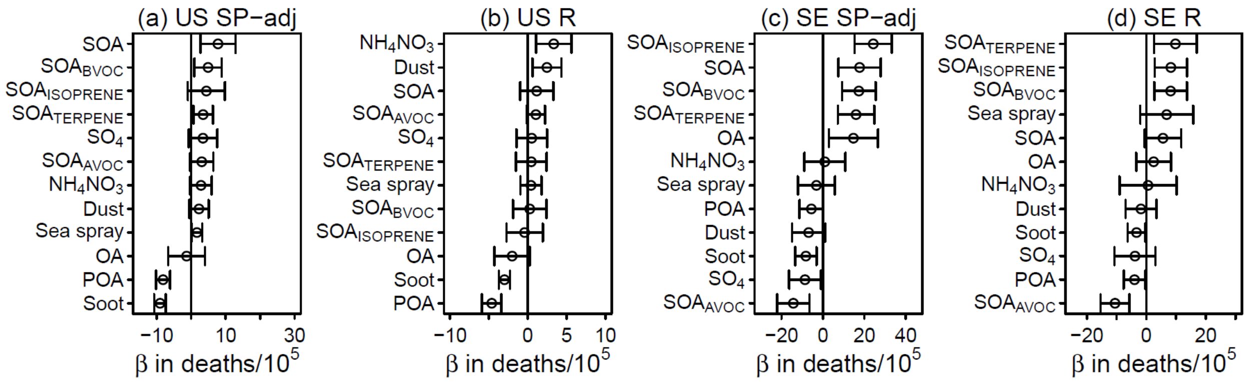

Supplementary Figure 5: Association of $PM_{2.5}$ SOA subcomponents with death rates across the contiguous U.S. (n=2,708) in black and southeastern (SE) U.S. (n=646) in red (open symbol) determined via regressed coefficients (β) from multiple linear regression and their 95% confidence intervals (whiskers). Model forms are single pollutant (SP, circles) and multipollutant for the entire $PM_{2.5}$ composition with refinement of SOA subcomponents into $SOA_{ISOPRENE}$, $SOA_{TERPENE}$, and SOA_{AVOC} (MP, triangles; See Supplementary Note). Regressed coefficients correspond to IQR-normalized species concentrations in units of deaths per 100,000 in population.

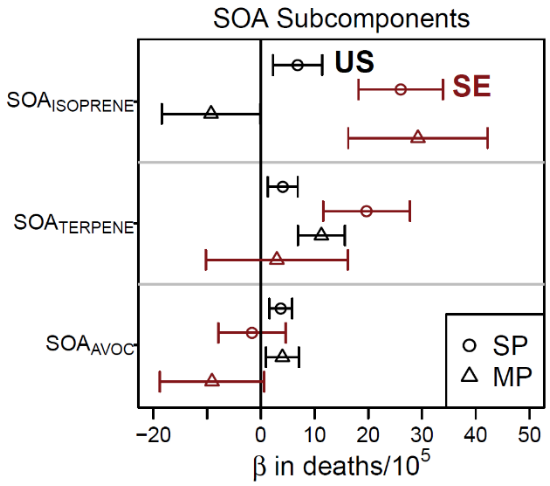

Supplementary Figure 6: AQS-observed and CMAQ-modeled $PM_{2.5}$ and its major components across the US for 2016. Here, dust is evaluated as Ca + Fe + Si + Ti + Mn + Al. Boxplots indicate the 2.5 to 97.5 percentiles (whiskers), interquartile range (shaded box), and median (horizontal line). The number of data points by species are: Total $PM_{2.5}$ 400,319; OC 28,422; Dust 22,129; SO_4 29,976; NH_4NO_3 29,726; Soot 27,311, Sea Spray 21,030.

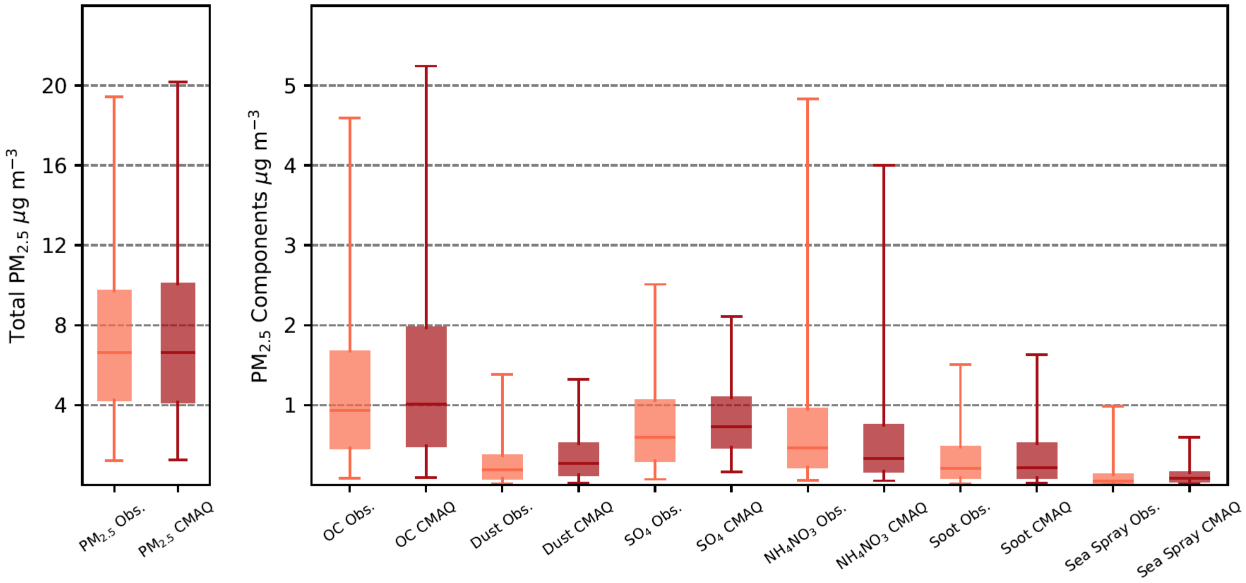

References

1 Appel, K. W. *et al.* The Community Multiscale Air Quality (CMAQ) model versions 5.3 and 5.3.1: system updates and evaluation. *Geosci. Model Dev.* **14**, 2867-2897, doi:10.5194/gmd-14-2867-2021 (2021).

2 Emery, C. *et al.* Recommendations on statistics and benchmarks to assess photochemical model performance. *J. Air Waste Manage. Assoc.* **67**, 582-598, doi:10.1080/10962247.2016.1265027 (2017).

3 Boylan, J. W. & Russell, A. G. PM and light extinction model performance metrics, goals, and criteria for three-dimensional air quality models. *Atmos. Environ.* **40**, 4946-4959, doi:10.1016/j.atmosenv.2005.09.087 (2006).

4 Pye, H. O. T. *et al.* Epoxide pathways improve model predictions of isoprene markers and reveal key role of acidity in aerosol formation. *Environ. Sci. Technol.* **47**, 11056-11064, doi:10.1021/es402106h (2013).

5 Karambelas, A., Pye, H. O. T., Budisulistiorini, S. H., Surratt, J. D. & Pinder, R. W. Contribution of isoprene epoxydiol to urban organic aerosol: Evidence from modeling and measurements. *Environ. Sci. Tech. Lett.* **1**, 278-283, doi:10.1021/ez5001353 (2014).

6 Budisulistiorini, S. H. *et al.* Simulating aqueous-phase isoprene-epoxydiol (IEPOX) secondary organic aerosol production during the 2013 Southern Oxidant and Aerosol Study (SOAS). *Environ. Sci. Technol.* **51**, 5026-5034, doi:10.1021/acs.est.6b05750 (2017).

7 Pye, H. O. T. *et al.* On the implications of aerosol liquid water and phase separation for organic aerosol mass. *Atmos. Chem. Phys.* **17**, 343-369, doi:10.5194/acp-17-343-2017 (2017).

8 Pye, H. O. T. *et al.* Modeling the current and future roles of particulate organic nitrates in the southeastern United States. *Environ. Sci. Technol.* **49**, 14195-14203, doi:10.1021/acs.est.5b03738 (2015).

9 Murphy, B. N. *et al.* Semivolatile POA and parameterized total combustion SOA in CMAQv5.2: impacts on source strength and partitioning. *Atmos. Chem. Phys.* **17**, 11107-11133, doi:10.5194/acp-17-11107-2017 (2017).

10 Liu, J. *et al.* Regional similarities and NOx-related increases in biogenic secondary organic aerosol in summertime southeastern United States. *J. Geophys. Res.-Atmos.* **123**, 10,620-610,636, doi:10.1029/2018JD028491 (2018).

11 Carlton, A. G. *et al.* Model representation of secondary organic aerosol in CMAQv4.7. *Environ. Sci. Technol.* **44**, 8553-8560, doi:10.1021/es100636q (2010).

12 Zhang, H. *et al.* Monoterpenes are the largest source of summertime organic aerosol in the southeastern United States. *P. Natl. Acad. Sci. USA* **115**, 2038, doi:10.1073/pnas.1717513115 (2018).

13 Pye, H. O. T. *et al.* Anthropogenic enhancements to production of highly oxygenated molecules from autoxidation. *P. Natl. Acad. Sci. USA* **116**, 6641, doi:10.1073/pnas.1810774116 (2019).

14 Xu, L. *et al.* Experimental and model estimates of the contributions from biogenic monoterpenes and sesquiterpenes to secondary organic aerosol in the southeastern United States. *Atmos. Chem. Phys.* **18**, 12613-12637, doi:10.5194/acp-18-12613-2018 (2018).

15 Lee, B. H. *et al.* Resolving ambient organic aerosol formation and aging pathways with simultaneous molecular composition and volatility observations. *ACS Earth Space Chem.* **4**, 391-402, doi:10.1021/acsearthspacechem.9b00302 (2020).
