# Supplementary material for: Secondary organic aerosol association with cardiorespiratory disease mortality in the United States
Source: Nat Commun. 2021 Dec 16;12:7215. doi: 10.1038/s41467-021-27484-1 (PMC8677800; doi:10.1038/s41467-021-27484-1)
Supplement: Supplementary file 2 — Reporting Summary [file 41467_2021_27484_MOESM2_ESM.pdf]

nature research

Corresponding author(s): Havala Pye

Last updated by author(s): 18 October 2021

Reporting Summary

Nature Research wishes to improve the reproducibility of the work that we publish. This form provides structure for consistency and transparency in reporting. For further information on Nature Research policies, see our Editorial Policies and the Editorial Policy Checklist.

Statistics

For all statistical analyses, confirm that the following items are present in the figure legend, table legend, main text, or Methods section.

n/a	Confirmed	
☐	☒	The exact sample size (*n*) for each experimental group/condition, given as a discrete number and unit of measurement
☒	☐	A statement on whether measurements were taken from distinct samples or whether the same sample was measured repeatedly
☒	☐	The statistical test(s) used AND whether they are one- or two-sided<br>*Only common tests should be described solely by name; describe more complex techniques in the Methods section.*
☐	☒	A description of all covariates tested
☐	☒	A description of any assumptions or corrections, such as tests of normality and adjustment for multiple comparisons
☐	☒	A full description of the statistical parameters including central tendency (e.g. means) or other basic estimates (e.g. regression coefficient) AND variation (e.g. standard deviation) or associated estimates of uncertainty (e.g. confidence intervals)
☒	☐	For null hypothesis testing, the test statistic (e.g. *F*, *t*, *r*) with confidence intervals, effect sizes, degrees of freedom and *P* value noted<br>*Give P values as exact values whenever suitable.*
☒	☐	For Bayesian analysis, information on the choice of priors and Markov chain Monte Carlo settings
☒	☐	For hierarchical and complex designs, identification of the appropriate level for tests and full reporting of outcomes
☒	☐	Estimates of effect sizes (e.g. Cohen's *d*, Pearson's *r*), indicating how they were calculated

Our web collection on statistics for biologists contains articles on many of the points above.

Software and code

Policy information about availability of computer code

Data collection	No software was used for data collection. CMAQv5.3.1 was used with WRFv4.1.1 meteorology to produce simulated air quality in previous work. CMAQ v5.3.1 is available at http://doi.org/10.5281/zenodo.3585898.
Data analysis	All statistical models used R v4.0.0. The analysis code for post-processing CMAQ predictions, aggregating CMAQ to annual-average component values, aligning data by county, and performing the statistical analyses have been deposited at https://doi.org/10.23719/1519254.

For manuscripts utilizing custom algorithms or software that are central to the research but not yet described in published literature, software must be made available to editors and reviewers. We strongly encourage code deposition in a community repository (e.g. GitHub). See the Nature Research guidelines for submitting code & software for further information.

Data

Policy information about availability of data

All manuscripts must include a data availability statement. This statement should provide the following information, where applicable:

- Accession codes, unique identifiers, or web links for publicly available datasets
- A list of figures that have associated raw data
- A description of any restrictions on data availability

The complete set of county-level concentrations and multiple regression results generated in this study have been deposited at data.gov under https://doi.org/10.23719/1519254. Mortality rate data used in this work are available from the Centers for Disease Control and Prevention, National Center for Health Statistics, CDC WONDER Online Database, released June 2017. Data are from the Compressed Mortality File 1999-2016 Series 20 No. 2U, 2016, as compiled from data provided by the 57 vital statistics jurisdictions through the Vital Statistics Cooperative Program. Last accessed at http://wonder.cdc.gov/cmf-icd10.html on Aug 31, 2020 9:15:06 AM. County Health Rankings 2018 data developed as a collaboration between the Robert Wood Johnson Foundation and the University of

Wisconsin Population Health Institute were obtained from https://www.countyhealthrankings.org/explore-health-rankings/rankings-data-documentation (last access: 31 March 2020). Observed air quality data is available from the EPA Air Quality System (AQS) available at https://www.epa.gov/aqs (last access: 18 October 2021). Source data for data generated in this work are provided with this paper.

Field-specific reporting

Please select the one below that is the best fit for your research. If you are not sure, read the appropriate sections before making your selection.

☐ Life sciences ☐ Behavioural & social sciences ☒ Ecological, evolutionary & environmental sciences

For a reference copy of the document with all sections, see nature.com/documents/nr-reporting-summary-flat.pdf

Ecological, evolutionary & environmental sciences study design

All studies must disclose on these points even when the disclosure is negative.

Study description	We used statistical models to associate observed cardiorespiratory mortality with fine particle air pollution components. Primary analyses use multiple linear regression to associate PM2.5 with cardiorespiratory mortality while adjusting for confounders. Spline fits in generalized additive models were used as a sensitivity analysis. Statistical associations were performed using observed cardiorespiratory mortality for the contiguous U.S. (2708 counties) and southeastern U.S. (646 counties).
Research sample	We used observed cardiorespiratory mortality from the CDC (see data availability) for the U.S. population by county. In addition, we used the publicly available CMAQ Model to predict concentrations of PM2.5 components. CMAQ provides full PM2.5 speciation that is not available via observations.
Sampling strategy	CMAQ simulations are computationally intensive and not continuously available for all years. CMAQ predictions were available for the contiguous U.S. for year 2016 from previous work which dictated the period of study. CDC data was publicly available on an annual basis.
Data collection	Observed mortality data was obtained from the CDC via the CDC WONDER Online Database. CMAQ simulations were used to produce air quality data. Simulations of air quality via CMAQ were conducted as part of previous work but aggregated to new species in this work.
Timing and spatial scale	Analysis was for data for the full year 2016, January 1 to December 31, annually averaged or aggregated. CMAQ's spatial domain was the contiguous U.S. The southeastern U.S. was examined as a sub-domain. CMAQ-predicted hourly concentrations were aggregated to an annual average. CDC cardiorespiratory mortality was aggregated for the year 2016.
Data exclusions	Counties were excluded if CDC did not provide data or confounders were not available for that county.
Reproducibility	We used multiple linear regression models (see Table 2) as well as a generalized additive model in the supplement. All model forms gave consistent information regarding our primary message about the importance of SOA. Since the cardiorespiratory mortality data was observed, there was no replication of the input data.
Randomization	NA-Cardiorespiratory mortality was observed. There was no intervention implemented by this study.
Blinding	NA-Cardiorespiratory mortality was observed. There was no intervention implemented by this study.

Did the study involve field work? ☐ Yes ☒ No

Reporting for specific materials, systems and methods

We require information from authors about some types of materials, experimental systems and methods used in many studies. Here, indicate whether each material, system or method listed is relevant to your study. If you are not sure if a list item applies to your research, read the appropriate section before selecting a response.

Materials & experimental systems

n/a	Involved in the study
☒	☐ Antibodies
☒	☐ Eukaryotic cell lines
☒	☐ Palaeontology and archaeology
☒	☐ Animals and other organisms
☒	☐ Human research participants
☒	☐ Clinical data
☒	☐ Dual use research of concern

Methods

n/a	Involved in the study
☒	☐ ChIP-seq
☒	☐ Flow cytometry
☒	☐ MRI-based neuroimaging
